# Relationship between DXA measured metrics of adiposity and glucose homeostasis; An analysis of the NHANES data

**DOI:** 10.1371/journal.pone.0216900

**Published:** 2019-05-22

**Authors:** Prasanna Santhanam, Steven P. Rowe, Jenny Pena Dias, Rexford S. Ahima

**Affiliations:** 1 Division of Endocrinology, Diabetes, & Metabolism, Department of Medicine, Johns Hopkins University School of Medicine, Baltimore, MD, United States of America; 2 The Russell H. Morgan Department of Radiology and Radiological Science, Johns Hopkins University School of Medicine, Baltimore, MD, United States of America; University of Bahrain, BAHRAIN

## Abstract

**Introduction:**

Obesity is associated with insulin resistance and type 2 diabetes. Dual-energy X-ray absorptiometry (DXA) is a means of determining body composition and body fat distribution at different sites including whole body and trunk–locations where there tends to be high correlation at an individual level.

**Methods:**

We performed an analysis of DXA-derived metrics of adiposity (truncal fat %,subtotal fat % and total fat %) from the NHANES database and then correlated the findings with markers of insulin resistance. We analyzed the data from DXA scans in NHANES 1999–2004. Homeostatic model assessment-insulin resistance and HOMA-β (beta-cell function) were estimated. Spearman correlation coefficients were calculated (ρ) between HOMA-IR,HOMA-β and different measures of obesity (Waist circumference(in cm), Body Mass Index (kg/m2), truncal fat %, subtotal fat % as well as total fat %) to gauge the relationship between markers of glucose homeostasis and DXA derived metrics of obesity. We also performed logarithmic transformation of HOMA-IR as well as HOMA-β to ensure normality of distribution and to meet the criteria for regression analysis. A forward selection model (by outcome and gender) was performed to predict log transformed insulin resistance (log HOMA-IR) as well as log transformed HOMA-β (log HOMA-β,measure of beta cell function) from age, serum triglycerides, HDL, trunk fat % and the SBP (in both males and females separately), after reviewing the spearman correlation coefficients.

**Results:**

There were a total of 6147 men and 6369 women who were part of the study cohort. There was a positive correlation between markers of adiposity and log HOMA-IR and log HOMA-β in both males and females.Truncal fat % had the highest nonparametric correlation coefficent with log HOMA-IR among the DXA derived fat% (0.54 in males and 048 in females). In the multivariate analysis, truncal fat % was an independent predictor of logHOMA-IR as well as logHOMA-β. In males, the significant predictors of log HOMA-IR were; age, truncal fat % and HDL cholesterol (Adjusted R square of 0.325 (±0.66), F(3,207) = 34.63, p < .01). In females, the significant predictors of log HOMA-IR were; age, truncal fat %, SBP, Serum triglyceride and HDL cholesterol (Adjusted R square of 0.307 (±0.65),F(5,198) = 18.9, p < .01). In both males and females, the significant predictors of log HOMA-β were; age, and truncal fat % (Males; adjusted R square of 0.25 (±0.63), F (2,208) = 36.4, p < .01, Females; adjusted R square of 0.27 (±0.62), F (2,201) = 38.4, p < .01).

**Conclusions:**

Body fat % on DXA is an imaging biomarker for insulin resistance. Incorporating this important information into DXA acquisitions and reporting frameworks may allow for this information to be available to providers who refer patients for these imaging studies.

## Introduction

Obesity imposes enormous economic burden on society and is associated with significant morbidity and mortality [1]. Traditionally, the degree of obesity has been classified by increasing body mass index (BMI). However, BMI is an overly simplistic tool at the population level and may not be an accurate marker of obesity. Dual-energy X-ray absorptiometry (DXA) is a quick and reliable way to accurately measure body composition [2, 3]. Body adiposity measured by DXA is associated with cardiometabolic risk even after adjustment for age, BMI, and waist circumference in the NHANES (National Health and Nutrition Examination Survey) population-based studies [4]. Given that DXA scans are routinely ordered by physicians in the primary care setting, any ability to utilize the information from these imaging studies to provide prognostic information about patients or to target focused work-ups for other conditions could prove to be of tremendous value.

Obesity is linked to insulin resistance and the development of type 2 diabetes. In adolescents, fasting insulin (in Mu/L) and waist circumference (WC) have most strongly been associated with parameters of obesity and homeostatic model assessment-insulin resistance (HOMA-IR) levels have been shown to be increased with increase in BMI and within each BMI category—higher body fat was associated with higher HOMA-IR [5, 6]. In a study involving chinese adults, Visceral Adiposity Index (VAI) (a mathematical equation used to describe adiposity dysfunction using triglycerides,BMI,WC and HDL) has been independently associated with HOMA-IR, after adjusting for other covariates in both males and females[7, 8].

We performed an analysis of DXA derived metrics of adiposity from the NHANES database and then correlated the findings with markers of insulin resistance.

## Methods

This was a cross sectional study with no repeated measures.

We analyzed the data from DXA scans in NHANES 1999–2004 that were acquired per manufacturer recommendations of the QDR 4500A fan beam densitometer (Hologic, Inc., Bedford, MA, USA) for measurement of body composition without performing additional imputational analysis [9]. The findings were then validated and recaliberated based on a study by Schoeller, et al. prior to release [10]. From the NHANES data, each set of measured and imputed values (prior imputed values computed by the CDC) can be merged with other data from NHANES to create analytic datasets (https://wwwn.cdc.gov/Nchs/Nhanes/Dxa/Dxa.aspx).

In the NHANES data, the Diabetes Diagnostic Laboratory at the University of Missouri in Columbia measured plasma glucose, serum C-peptide and insulin on participants, aged 12 years and older and this data was compiled [9]. The data for the variables—age (years), WC(cm), BMI (kg/m^2^), average systolic blood pressure (SBP, mm/Hg), serum triglycerides (mg/dl), LDL (mg/dl), and fasting insulin level (s) (μU/ml) were tabulated.

The HOMA-IR and the HOMA-β (or HOMA-B)(beta-cell function) were estimated, as has been previously described [11, 12]. The formula used was HOMA-IR = (plasma glucose x insulin)/405 (glucose in mg/dl), and HOMA-β = 360 x insulin/ (glucose-63) % (glucose in mg/dl). We gathered the fat %for the following adiposity metrics as obtained from DXA; truncal fat %, subtotal fat %(total body fat %—head fat %(measured from the head alone) as well as total body fat % for both males and females.Since men and women have different body fat distribution, those two groups were analyzed separately.

### Statistical analysis

We performed descriptive statistics on the variables. Additional imputational analysis was not performed since the NHANES data had prior imputed values. We tabulated the results and used % body fat for the regression analysis. Though % body fat indices may not be indicated of visceral obesity or even fat distribution, they do not need to be adjusted with respect to BMI and muscle mass (as opposed to absolute truncal fat,subtotal fat etc).

Spearman correlation coefficients were first calculated (ρ) between HOMA-IR,HOMA-β and different measures of obesity (WC(in cm), BMI (kg/m2), truncal fat%, subtotal fat %as well as total fat %)to gauge the relationship between markers of glucose homeostasis and DXA derived indices of obesity. We first perfomed the Kolmogorov-Smirnov test and qualitative inspection by mean and median, kurtosis, skewness. We also plotted the Q-Q plots and it is shown in **S1 File**. The outliers,data with negative values,missing data were noted and removed prior to statistical analysis.Since both HOMA-IR and HOMA-β failed the normality test, we performed a logarithmic transformation of HOMA-IR as well as HOMA-β to ensure normality of distribution and to meet the criteria for regression analysis. The log transformed HOMAIR and HOMA-β showed a normal distribution on visual inspection.

A forward model selections (by outcome and gender)was performed to predict log transformed insulin resistance (log HOMA-IR) as well as log transformed HOMA-β (log HOMA-β,measure of beta cell function)from age, serum triglycerides, HDL, trunk fat % and the SBP (in both males and females separately), after reviewing the spearman correlation coefficients. The log HOMA-IR and log HOMA-β were the independent outcome variables and age,serum triglyceride,HDL cholesterol and truncal fat % were the predictors. These variables were used since they are the components of the metabolic syndrome and are generally associated with cardiovascular outcomes.SPSS 25 (IBM, Armonk, NY, USA) was used for statistical analysis.The data is shown in the SPSS format as **S1 Dataset.**

## Results

There were a total of 6147 men and 6369 women who were included in the study based on the available data. The baseline data is tabulated in **Table 1**.

**Table 1:** Descriptive statistics of the study cohort.

		Males			Females	
	N	Mean	SD	N	Mean	SD
Age at Screening (years)	6147	38.78	22.44	6369	38.74	22.14
WC (cm)	5993	93.69	17.33	6174	91.47	16.55
BMI(kg/m2)	6048	26.54	6.18	6250	27.51	7.06
SBP (mm/Hg)	762	123.21	17.11	743	119.40	21.14
DBP(mm/Hg)	762	66.12	16.70	743	66.96	13.06
Truncal Fat %	5874	26.58	8.87	5186	36.80	9.21
Subtotal Fat %	5874	26.82	7.67	5186	39.73	7.66
Total Percent Fat	5874	26.68	7.21	5186	38.77	7.33
Fasting Glucose (mg/dL)	6147	103.34	31.75	6369	98.41	30.27
Insulin (uU/mL)	6147	12.99	12.61	6369	13.27	13.11
LDL-chol (mg/dL)	4389	111.60	36.83	4667	111.35	35.61
HDL-chol (mg/dL)	1438	46.29	12.30	1528	54.07	14.76
Triglyceride (mg/dL)	4756	136.06	136.98	4997	126.11	88.24

SBP = Systolic Blood Pressure, N = Sample Size, BMI = Body Mass Index, WC = Waist Circumference

The non-parametric correlation coefficients (spearman(ρ)) between these DXA derived metrics of obesity (truncal fat %,subtotal fat % as well as total fat %), BMI and WC and HOMA-IR and HOMA-β are shown in **Table 2.**

**Table 2:** Nonparametric correlation coefficients between log transformed HOMA-IR and HOMA-β.

N			log HOMA-IR	log HOMA-β
Male	Subtotal Fat %	5874	0.533**	0.313**
	Truncal Fat %	5874	0.543**	0.285**
	Total Fat %	5874	0.534**	0.314**
	Waist Circumference (cm)	5993	0.503**	0.236**
	BMI (kg/m2)	6048	0.524**	0.319**
Female	Subtotal Fat %	5186	0.414**	0.157**
	Truncal Fat %	5186	0.483**	0.193**
	Total Fat %	5186	0.418**	0.160**
	WC (cm)	6174	0.462**	0.211**
	BMI(kg/m2)	6369	0.483**	0.251**

N = sample size, significant results (p <0.01) are marked with **

**Fig 1:**
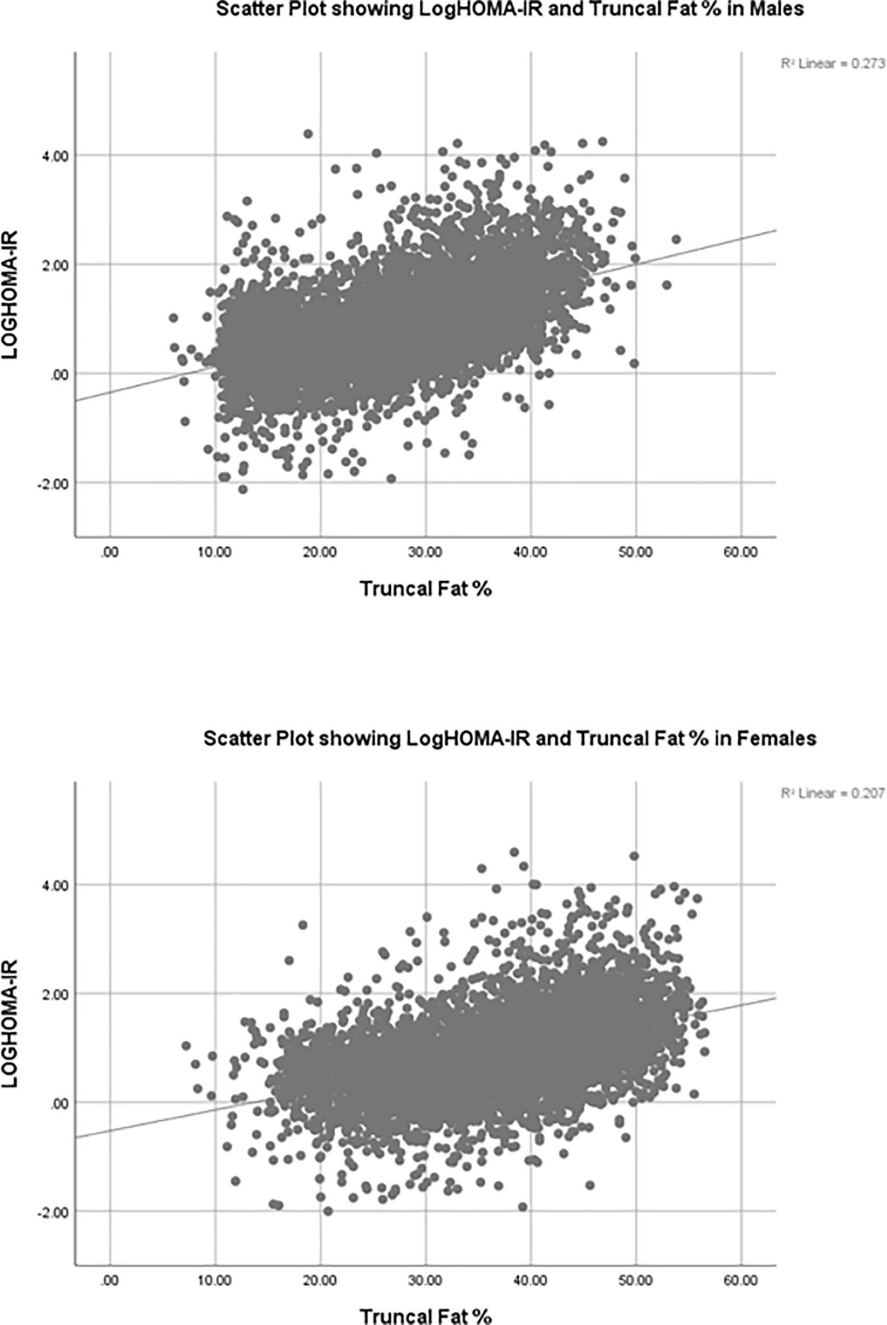
The scatter plots outlining the relationship between log HOMA-IR and trucal fat % in males and females).

**Fig 2:**
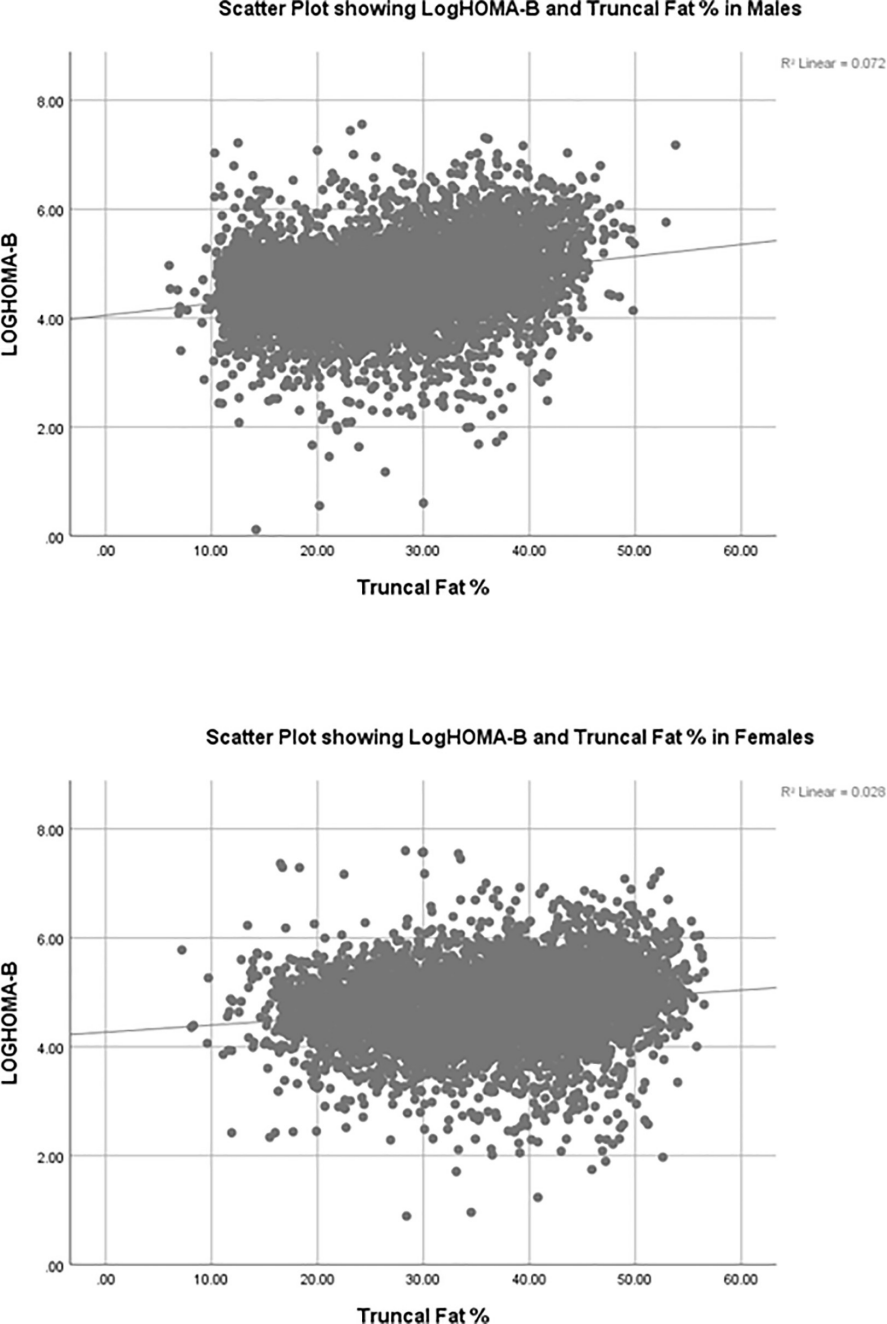
The scatter plots outlining the relationship between log HOMA-β and trucal fat % in males and females.

All the variables correlated significantly with logHOMA-IR and logHOMA-β. Truncal fat % had the highest correlation coefficient with log HOMA-IR among all the obesity metrics in both males (spearman rho (ρ) = 0.543, p <0.01) and females (ρ = 0.483, p <0.01). The scatter plots are shown in **Fig 1 and Fig 2.**

The forward step multiple regression was used to predict log HOMAIR and log HOMA-β from Truncal fat %, age, serum triglyceride, HDL cholesterole and average systolic blood pressure(SBP) in both men and women since, they are components of metabolic syndrome. There was linearity, independence of residuals and homoscedasticity, as assessed by visual inspection of a plot of studentized residuals versus unstandardized predicted values. Outliers,high leverage points were removed to account for unusual high points like HOMA-IR and HOMA-β associated with severe high insulin levels and other errors in data collection.The residuals were normally distributed.There was no evidence of multicollinearity, as assessed by tolerance values greater than 0.1. The assumption of normality was met, as assessed by a visual Q-Q Plot. In males, the significant predictors of log HOMA-IR were; age, truncal fat % and HDL cholesterol. The model had an adjusted R square of 0.325 and a standard error of estimate of 0.66. The regression model statistically significantly predicted log HOMA-IR (F(3,207) = 34.63, p < .01).In females, the significant predictors of log HOMA-IR were; age, truncal fat %,SBP, Serum triglyceride and HDL cholesterol. The model had an adjusted R square of 0.307 and a standard error of estimate of 0.65.The regression model statistically significantly predicted log HOMA-IR (F (5,198) = 18.9, p < .01). In both males and females, the significant predictors of log HOMA-β were; age, and truncal fat %. In men, the model had an adjusted R square of 0.25 and a standard error of estimate of 0.63. The regression model statistically significantly predicted log HOMA-β (F (2,208) = 36.4, p < .01).In females, the model had an adjusted R square of 0.27 and a standard error of estimate of 0.62.The regression model statistically significantly predicted log HOMA-β (F (2,201) = 38.4, p < .01). Truncal fat % was a significant predictor of both log HOMA-IR and log HOMA-β after adjusting for the variables associated with metabolic syndrome. Age and Truncal fat % were the only significant predictors of both log HOMA-IR and log HOMA-β.The results are outlined in Tables (3–6).

**Table 3:** The results of the regression with log HOMA-IR as the outcome.

Males		Unstandardized Coefficients		Standardized	95.0% Confidence Interval for B		
		B	STD e	Beta	LL	UL	P Value
Model 1	(Constant)	-0.36	0.15		-0.65	-0.06	0.02
	Truncal Fat %	0.05	0.01	0.52	0.04	0.06	0.00
Model 2	(Constant)	-0.30	0.15		-0.59	-0.01	0.04
	Truncal Fat %	0.06	0.01	0.62	0.04	0.07	0.00
	Age	-0.01	0.00	-0.22	-0.01	0.00	0.00
Model 3	(Constant)	0.29	0.26		-0.21	0.80	0.26
	Truncal Fat %	0.05	0.01	0.57	0.04	0.06	0.00
	Age	-0.01	0.00	-0.19	-0.01	0.00	0.00
	HDL	-0.01	0.00	-0.17	-0.02	0.00	0.01
** **	**Females**						
Model 1	(Constant)	-0.54	0.20		-0.94	-0.14	0.01
	Truncal Fat %	0.04	0.01	0.45	0.03	0.05	0.00
Model 2	(Constant)	0.16	0.29		-0.41	0.74	0.57
	Truncal Fat %	0.04	0.01	0.42	0.03	0.05	0.00
	HDL	-0.01	0.00	-0.20	-0.02	0.00	0.00
Model 3	(Constant)	0.07	0.29		-0.50	0.64	0.81
	Truncal Fat %	0.04	0.01	0.48	0.03	0.05	0.00
	Age	-0.01	0.00	-0.15	-0.01	0.00	0.03
	HDL	-0.01	0.00	-0.17	-0.02	0.00	0.01
Model 4	(Constant)	-0.59	0.36		-1.30	0.12	0.10
	Truncal Fat %	0.04	0.01	0.45	0.03	0.05	0.00
	Age	-0.01	0.00	-0.28	-0.02	0.00	0.00
	HDL	-0.01	0.00	-0.21	-0.02	0.00	0.00
	SBP	0.01	0.00	0.24	0.00	0.01	0.00
Model 5	(Constant)	-0.77	0.36		-1.48	-0.06	0.03
	Truncal Fat %	0.04	0.01	0.42	0.02	0.05	0.00
	Age	-0.01	0.00	-0.33	-0.02	-0.01	0.00
	HDL	-0.01	0.00	-0.18	-0.02	0.00	0.01
	SBP	0.01	0.00	0.25	0.00	0.01	0.00
	Triglyceride	0.00	0.00	0.18	0.00	0.00	0.01

SBP: Systolic blood pressure, HDL: HDL cholesterol, STD e: standard error

**Table 4:** The collinearity diagnostics as well as R-square values for log HOMA-IR.

		Correlations			Collinearity Statistics		R Square	Adjusted R Square	STD e
Gender		Zero-order	Partial	Part	Tolerance	VIF			
Male	**Model 1**						0.27	0.27	0.68
	Truncal Fat	0.52	0.52	0.52	1.00	1.00			
	**Model 2**						0.31	0.30	0.67
	Truncal Fat	0.52	0.55	0.55	0.78	1.28			
	Age	0.08	-0.22	-0.19	0.78	1.28			
	**Model 3**						0.33	0.32	0.66
	Truncal Fat	0.52	0.51	0.48	0.72	1.40			
	Age	0.08	-0.20	-0.17	0.77	1.30			
	HDL	-0.31	-0.19	-0.16	0.92	1.09			
Female	**Model 1**						0.21	0.20	0.70
	Truncal Fat	0.45	0.45	0.45	1.00	1.00			
	**Model 2**						0.25	0.24	0.68
	Truncal Fat	0.45	0.43	0.41	0.97	1.03			
	HDL	-0.28	-0.23	-0.20	0.97	1.03			
	**Model 3**						0.27	0.25	0.68
	Truncal Fat	0.45	0.45	0.43	0.78	1.28			
	Age	0.02	-0.15	-0.13	0.80	1.26			
	HDL	-0.28	-0.19	-0.16	0.92	1.09			
	**Model 4**						0.30	0.28	0.66
	Truncal Fat	0.45	0.43	0.40	0.77	1.30			
	Age	0.02	-0.24	-0.21	0.56	1.79			
	HDL	-0.28	-0.23	-0.20	0.87	1.15			
	SBP	0.15	0.21	0.18	0.57	1.74			
	**Model 5**						0.32	0.31	0.65
	Truncal Fat	0.45	0.40	0.36	0.74	1.35			
	Age	0.02	-0.28	-0.24	0.53	1.87			
	HDL	-0.28	-0.20	-0.16	0.84	1.19			
	SBP	0.15	0.23	0.19	0.57	1.75			
	Triglyceride	0.29	0.19	0.16	0.83	1.20			

Truncal Fat (in %)

**Table 5:** The results of the regression with log HOMA-β as the outcome.

Gender			Unstandardized Coefficients		Standardized Coefficients	95.0% Confidence Interval for β		P Value
			β	SD Error	β	Lower Bound	Upper Bound	
Male	Model 1	(Constant)	4.03	0.15		3.73	4.33	0.00
		Truncal Fat %	0.02	0.01	0.27	0.01	0.03	
	Model 2	(Constant)	4.14	0.14		3.87	4.41	0.00
		Truncal Fat %	0.04	0.01	0.50	0.03	0.05	0.00
		Age	-0.02	0.00	-0.49	-0.02	-0.01	0.00
Female	Model 1	(Constant)	5.31	0.10		5.12	5.49	0.00
		Age	-0.01	0.00	-0.39	-0.02	-0.01	0.00
	Model 2	(Constant)	4.38	0.18		4.03	4.74	0.00
		Truncal Fat %	0.03	0.01	0.39	0.02	0.04	0.00
		Age	-0.02	0.00	-0.54	-0.02	-0.01	0.00

**Table 6:** Collinearity diagnostics and R-square values for log HOMA-β.

Gender			Correlations			Collinearity Statistics		R Square	Adj R Square	SD Error
			Zero-order	Partial	Part	Tolerance	VIF			
Male	Model 1	(Constant)						0.07	0.07	0.70
		Truncal Fat %	0.27	0.27	0.27	1.00	1.00			
	Model 2	(Constant)						0.26	0.25	0.63
		Truncal Fat %	0.27	0.46	0.44	0.78	1.28			
		Age	-0.26	-0.45	-0.43	0.78	1.28			
Female	Model 1	(Constant)						0.15	0.15	0.67
		Age	-0.39	-0.39	-0.39	1.00	1.00			
	Model 2	(Constant)						0.28	0.27	0.62
		Truncal Fat %	0.17	0.38	0.35	0.84	1.19			
		Age	-0.39	-0.51	-0.50	0.84	1.19			

Truncal Fat in %

## Discussion

The body fat distribution aspects of metabolic disease has been evaluated since early 1980s. In one of those early studies, it was shown that women with predominantly upper segment obesity and a significantly higher plasma glucose and insulin levels compared to lower segment [13].

The increased expression of markers like WNT1 inducible signaling pathway protein 2 (WISP2) leads to hypertrophic obesity in the human abdominal subcutaneous adipose tissue and this is associated with increased abdominal fat and insulin resistance[14]. DXA measured body fat % has been used as the ‘gold standard’ for developing indices like Body Adiposity Index (BAI) [15].In children adolescents, DXA has been used as the reference method to compare other methods of body fat % estimation like bioelectrical impedance analysis (BIA) technology and Slaughter skinfold-thickness equations[16]. DXA for body fat measurements has some limitations. For example, DXA scan measured visceral adipose tissue is most accurate using the GE iDXA recommended that participants have a BMI >/ = 25 kg/m(2), or VAT mass > 500 g according to one study[17]. There have been conflicting results on the use of DXA measured body fat % in certain subgroups like pubertal obese adolescents were other metrics like arm circumference in boys and BMI in girls were likely to be more helpful [18]. On the other hand, DXA is frequently used to measure changes in adiposity in interventional studies (like laprascopic gastric sleeve) where the main outcome is improvement is insulin resistance [19]

Our study has shown that DXA derived metrics of adiposity directly correlate with insulin resistance (i.e.HOMA-IR and HOMA-β) in both men and women. Truncal fat % is a predictor of log HOMA-IR as well as logHOMA-β in both men and women after adjusting for serum triglycerides,age,HDL and SBP. As such, body fat % measured by DXA may serve as an imaging biomarker for insulin resistance. Incorporating this important information into standardized DXA acquisitions and reporting frameworks may allow for additional high-value information to be available to providers who refer patients for these imaging studies.

Prior studies have demonstrated that some DXA measured body fat indices (from the NHANES data), especially higher truncal and total fat mass %, have correlated with increased cardiovascular death [20]. In another epidemiological study involving a large population, the DXA derived body fat %was independently associated with increased mortality [21]. DXA derived anthropometric measures have also shown that in women with PCOS (a condition associated with insulin resistance), truncal fat, WC and BMI were important predictors of HOMA-IR out of which truncal fat had the highest bivariate association [22].

There are limitations in our study. Firstly, a few high-leverage data points may be driving the linear fit and correlation and it is unclear why these drive the relationship in a positive direction. The data is cross sectional with respect to plasma glucose and insulin levels as well as the DXA measured fat %.

## Conclusion

Our study has shown a relationship between the body fat % and markers of insulin resistance in the NHANES population-based database. In the future, the DXA derived body composition may serve as a vital tool to analyze and examine adiposity given the ease of availability,low cost and standardized methodologies to interpret the results.

## Supporting information

S1 File The tests for normality including the Kolmogorov-Smirnov test and Q-Q plots. (PDF)

S1 Dataset The compiled data in an SPSS format. The merged SPSS files for the years in which there was data regarding glucose, insulin levels as well as body composition data.
(SAV)
