# Supplementary material for: Relationship between DXA measured metrics of adiposity and glucose homeostasis; An analysis of the NHANES data
Source: PLoS One. 2019 May 22;14(5):e0216900. doi: 10.1371/journal.pone.0216900 (PMC6530894; doi:10.1371/journal.pone.0216900)
Supplement: S1 File — (PDF) [file pone.0216900.s001.pdf]

```
EXAMINE VARIABLES=HOMAIR HOMAB
  /PLOT BOXPLOT HISTOGRAM NPPLOT
  /COMPARE GROUPS
  /STATISTICS DESCRIPTIVES EXTREME
  /CINTERVAL 95
  /MISSING LISTWISE
  /NOTOTAL.
```

Explore

Notes

Output Created		29-MAR-2019 11:56:46
Comments		
Input	Data	C:\Users\Prasanna\Desktop\PLOS REBUTTAL AND REVISION\PLOSANALYSIS2.sav
	Active Dataset	DataSet1
	Filter	<none>
	Weight	<none>
	Split File	<none>
	N of Rows in Working Data File	12581
Missing Value Handling	Definition of Missing	User-defined missing values for dependent variables are treated as missing.
	Cases Used	Statistics are based on cases with no missing values for any dependent variable or factor used.
Syntax		EXAMINE VARIABLES=HOMAIR HOMAB /PLOT BOXPLOT HISTOGRAM NPPLOT /COMPARE GROUPS /STATISTICS DESCRIPTIVES EXTREME /CINTERVAL 95 /MISSING LISTWISE /NOTOTAL.

Notes

Resources	Processor Time	00:00:05.13
	Elapsed Time	00:00:04.24

Case Processing Summary

	Cases					
	Valid		Missing		Total	
	N	Percent	N	Percent	N	Percent
HOMAIR	12561	99.8%	20	0.2%	12581	100.0%
HOMAB	12561	99.8%	20	0.2%	12581	100.0%

Descriptives

			Statistic	Std. Error
HOMAIR	Mean		3.5499	.04640
	95% Confidence Interval for Mean	Lower Bound	3.4589	
		Upper Bound	3.6409	
	5% Trimmed Mean		2.8439	
	Median		2.3177	
	Variance		27.046	
	Std. Deviation		5.20057	
	Minimum		.12	
	Maximum		136.28	
	Range		136.16	
	Interquartile Range		2.37	
	Skewness		8.866	.022
	Kurtosis		126.878	.044
HOMAB	Mean		134.1132	13.01142
	95% Confidence Interval for Mean	Lower Bound	108.6088	
		Upper Bound	159.6176	
	5% Trimmed Mean		128.7100	
	Median		110.6743	
	Variance		2126540.843	
	Std. Deviation		1458.26638	
	Minimum		-157464.00	
	Maximum		18828.00	
	Range		176292.00	
	Interquartile Range		102.99	

Descriptives

		Statistic	Std. Error
	Skewness	-100.284	.022
	Kurtosis	10868.960	.044

Extreme Values

			Case Number	Value
HOMAIR	Highest	1	90	136.28
		2	5605	115.30
		3	423	98.89
		4	9	97.06
		5	1258	92.14
	Lowest	1	10970	.12
		2	11589	.12
		3	12159	.13
		4	12352	.13
		5	12435	.14
HOMAB	Highest	1	1	18828.00
		2	2	16701.52
		3	3	15624.00
		4	4	5364.00
		5	5	4968.00
	Lowest	1	12561	-157464.00
		2	12560	-12488.40
		3	12559	-12088.80
		4	12558	-11106.00
		5	12557	-9097.20

Tests of Normality

	Kolmogorov-Smirnov[a]		
	Statistic	df	Sig.
HOMAIR	.262	12561	.000
HOMAB	.462	12561	.000

a. Lilliefors Significance Correction

HOMAIR

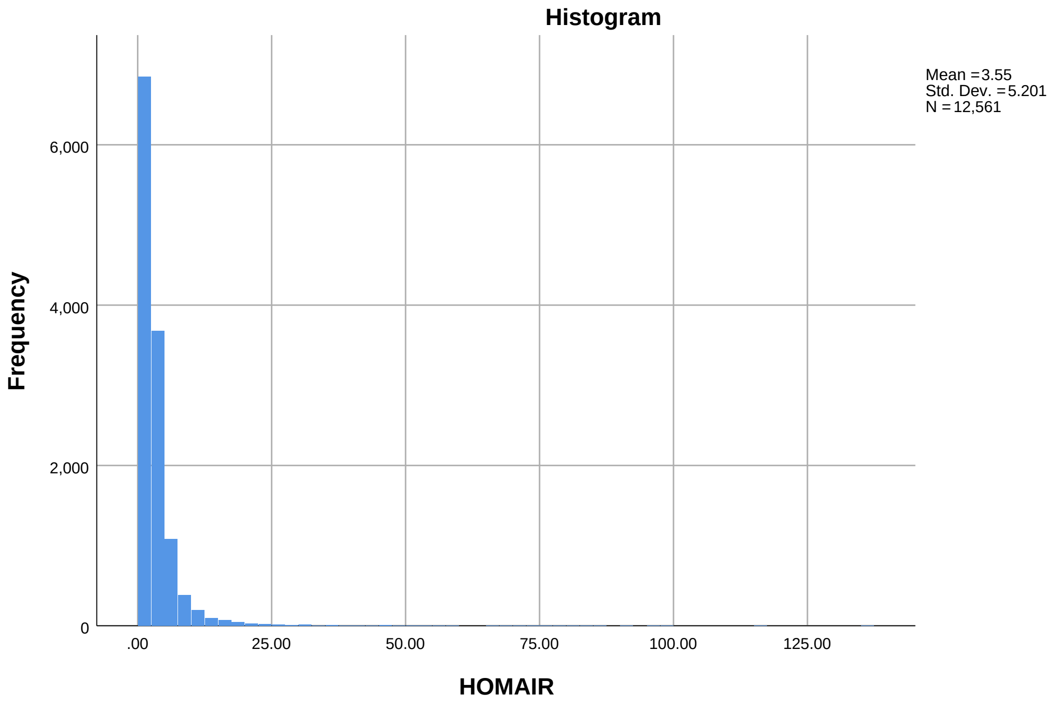

Histogram
Mean =3.55
Std. Dev. =5.201
N =12,561
Frequency
6,000
4,000
2,000
0
.00
25.00
50.00
75.00
100.00
125.00
HOMAIR

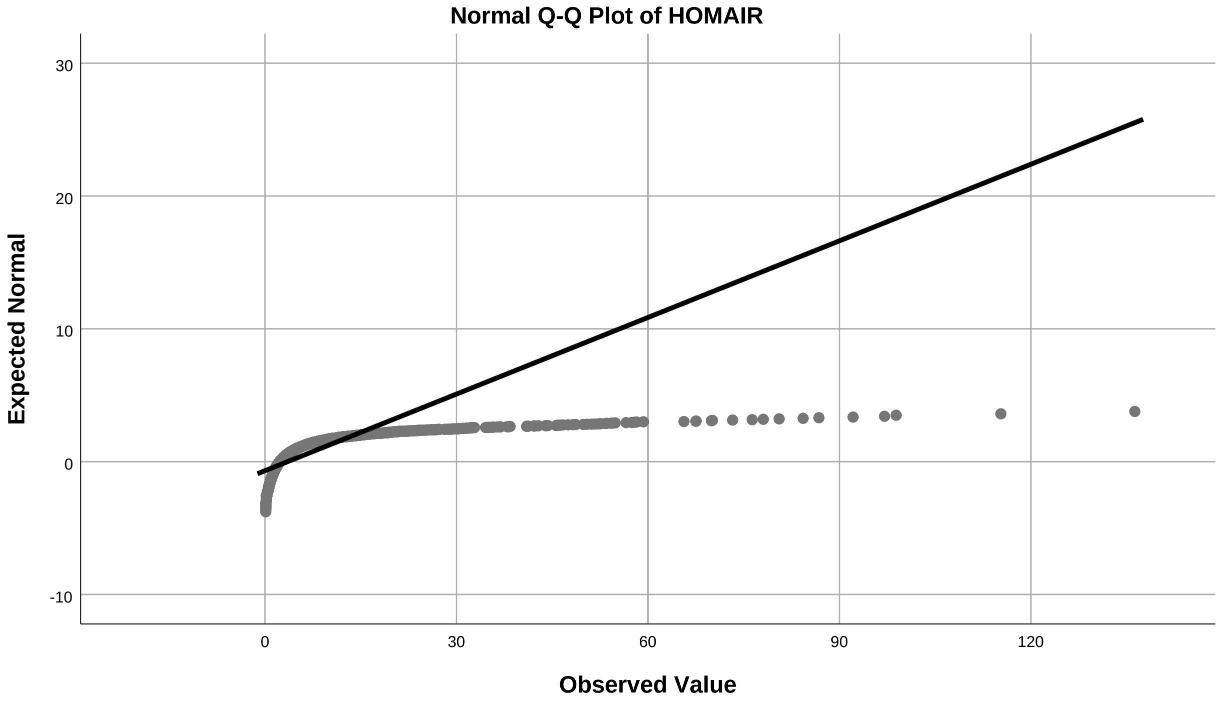

Normal Q-Q Plot of HOMAIR
Expected Normal
30
20
10
0
-10
0
30
60
90
120
Observed Value

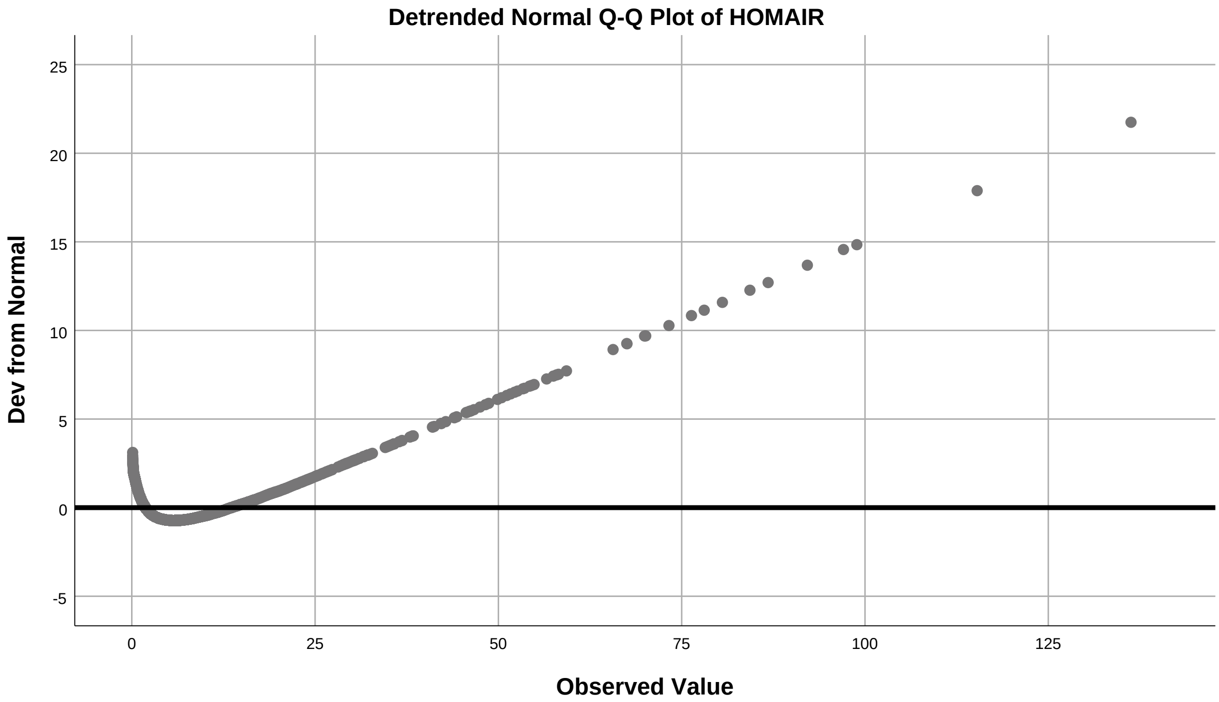

Detrended Normal Q-Q Plot of HOMAIR
Dev from Normal
Observed Value

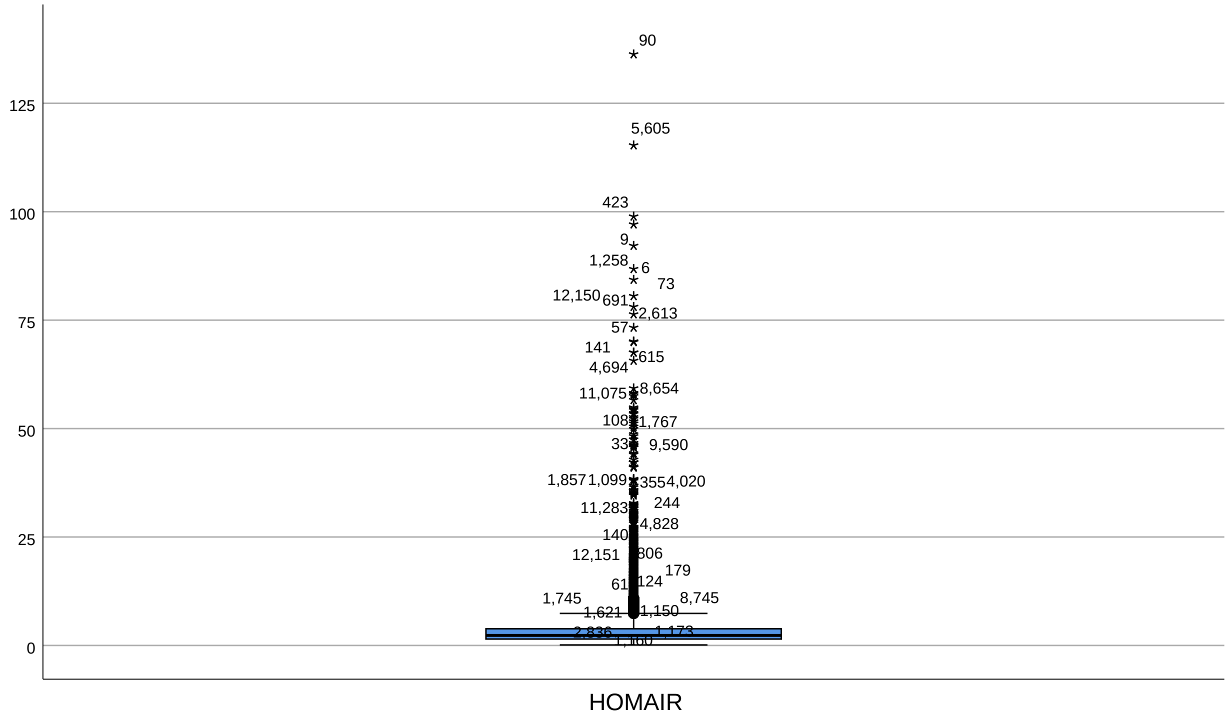

HOMAIR

HOMAB

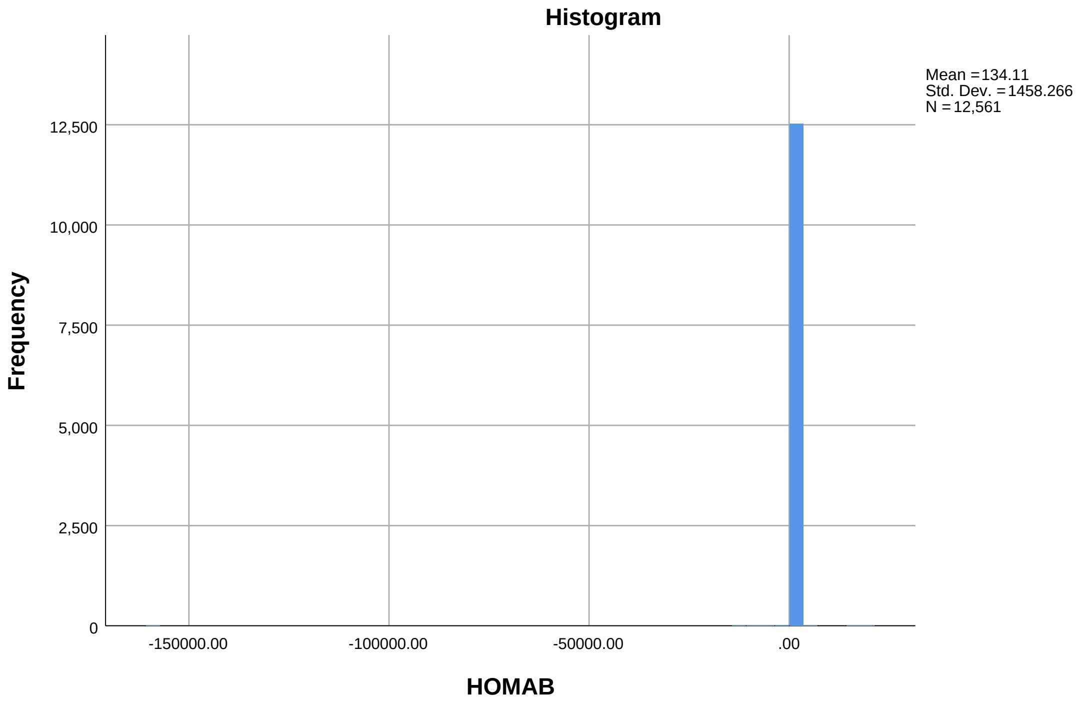
Histogram
Mean =134.11
Std. Dev. =1458.266
N =12,561
Frequency
12,500
10,000
7,500
5,000
2,500
0
-150000.00
-100000.00
-50000.00
.00
HOMAB

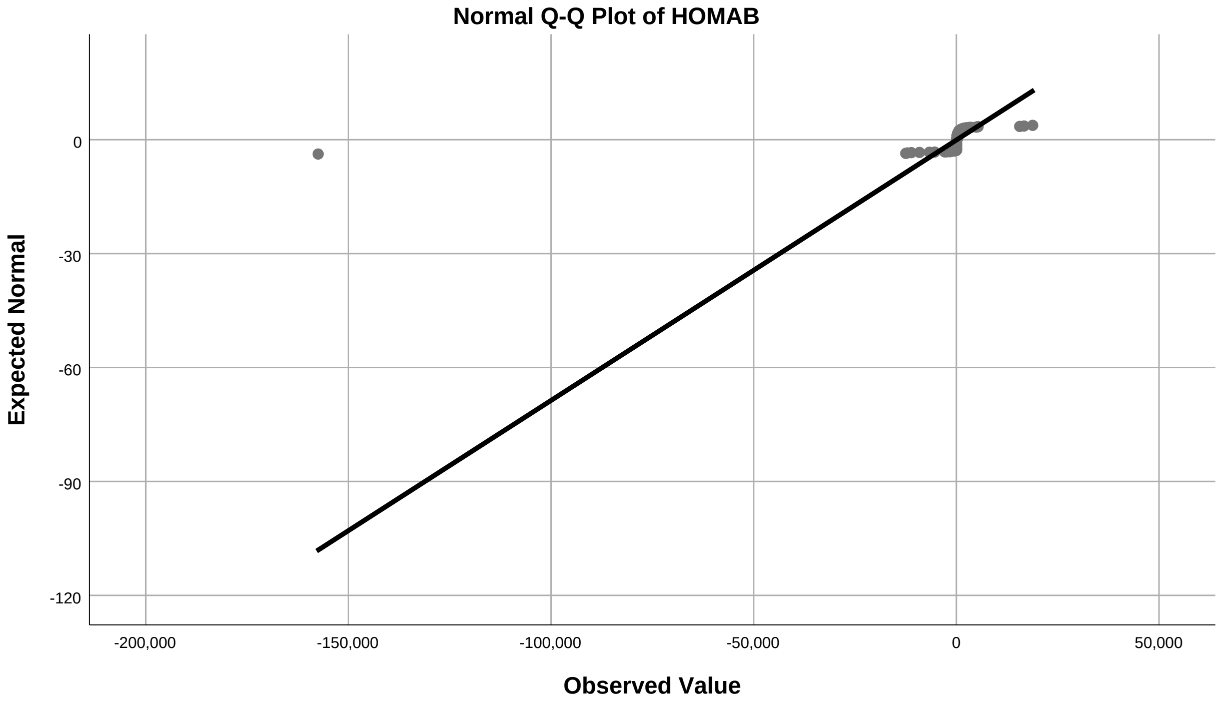
Normal Q-Q Plot of HOMAB
Expected Normal
0
-30
-60
-90
-120
-200,000
-150,000
-100,000
-50,000
0
50,000
Observed Value

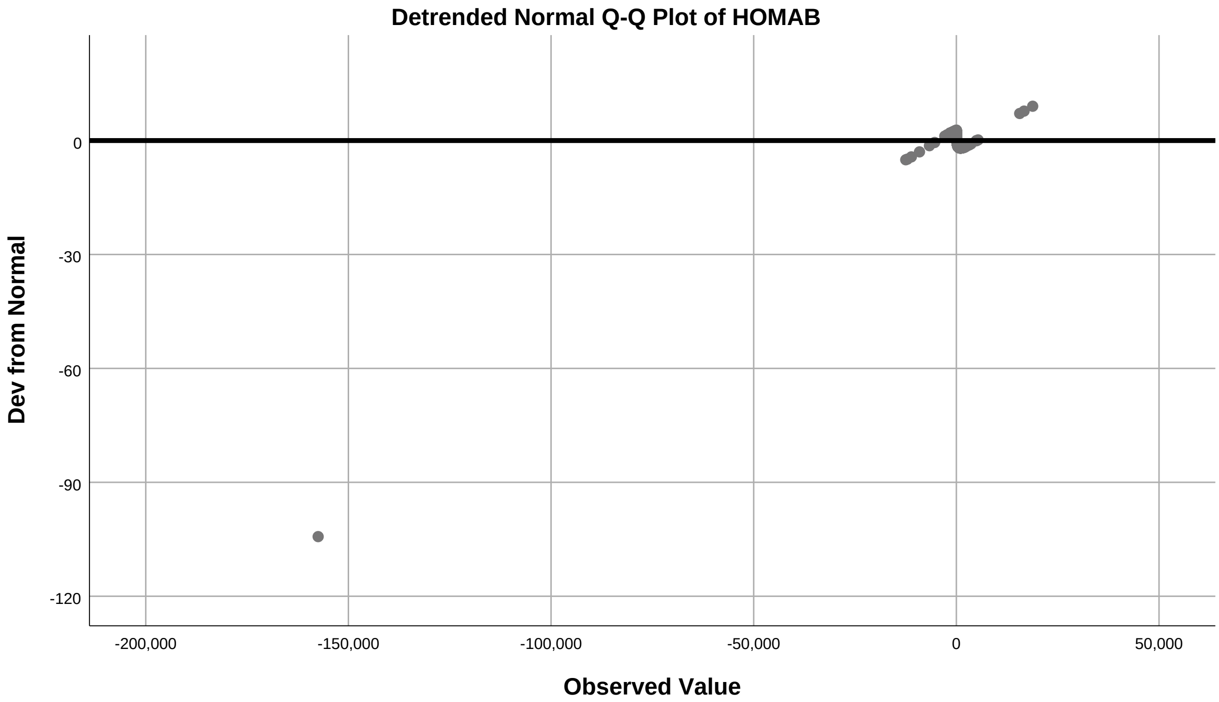
Detrended Normal Q-Q Plot of HOMAB
Dev from Normal
0
-30
-60
-90
-120
-200,000
-150,000
-100,000
-50,000
0
50,000
Observed Value

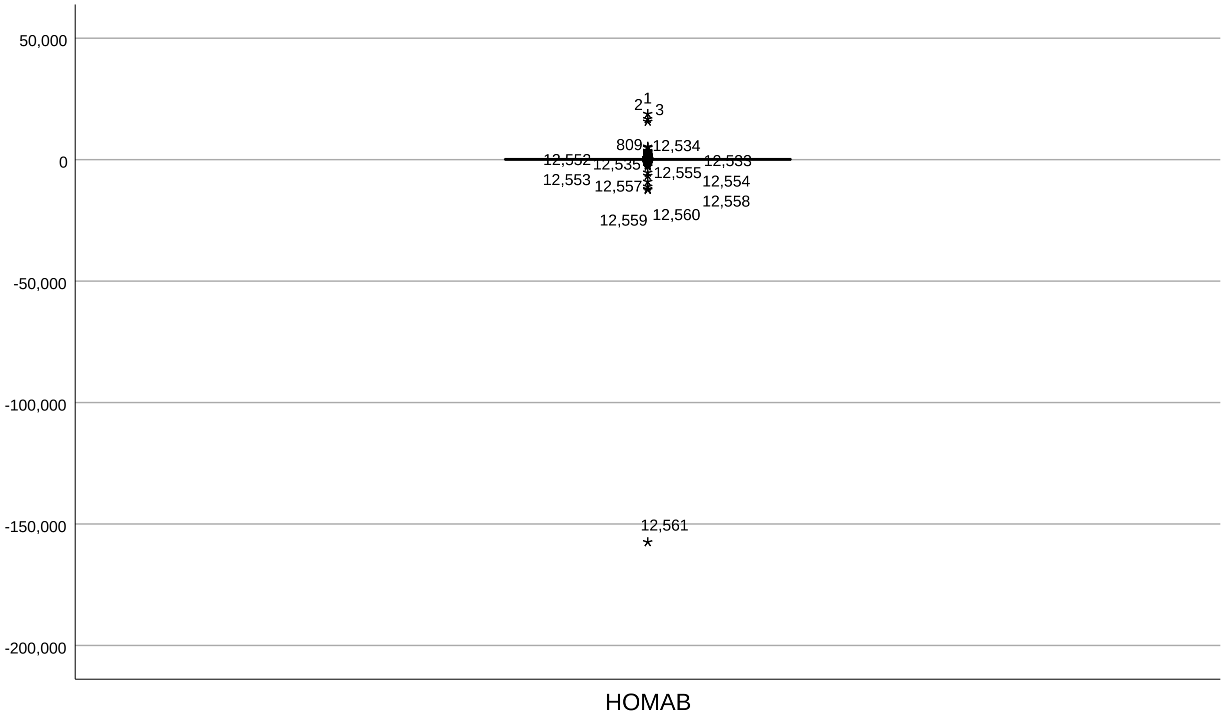
50,000
0
-50,000
-100,000
-150,000
-200,000
2
1
3
809
12,534
12,552
12,535
12,533
12,555
12,553
12,557
12,554
12,558
12,559
12,560
12,561
HOMAB

```
SAVE OUTFILE='C:\Users\Prasanna\Desktop\PLOS REBUTTAL  AND REVISION\PLOSANALYS
IS2a.sav'
  /COMPRESSED.
SORT CASES BY HOMAIR(D).
DATASET ACTIVATE DataSet1.

SAVE OUTFILE='C:\Users\Prasanna\Desktop\PLOS REBUTTAL  AND REVISION\PLOSANALYS
IS2a.sav'
  /COMPRESSED.
EXAMINE VARIABLES=HOMAIR HOMAB
  /PLOT BOXPLOT
  /COMPARE GROUPS
  /STATISTICS DESCRIPTIVES EXTREME
  /CINTERVAL 95
  /MISSING LISTWISE
  /NOTOTAL.
```

Explore

Notes

Output Created		29-MAR-2019 12:24:36
Comments		
Input	Data	C:\Users\Prasanna\Desktop\PLOS REBUTTAL AND REVISION\PLOSANALYSIS2a.sav
	Active Dataset	DataSet1
	Filter	<none>
	Weight	<none>
	Split File	<none>
	N of Rows in Working Data File	12565
Missing Value Handling	Definition of Missing	User-defined missing values for dependent variables are treated as missing.
	Cases Used	Statistics are based on cases with no missing values for any dependent variable or factor used.

Notes

Syntax		EXAMINE VARIABLES=HOMAIR HOMAB<br>/PLOT BOXPLOT<br>/COMPARE GROUPS<br>/STATISTICS DESCRIPTIVES EXTREME<br>/CINTERVAL 95<br>/MISSING LISTWISE<br>/NOTOTAL.
Resources	Processor Time	00:00:01.22
	Elapsed Time	00:00:01.07

```
 SORT CASES BY HOMAIR(A).
SORT CASES BY HOMAB(A).
DATASET ACTIVATE DataSet1.

SAVE OUTFILE='C:\Users\Prasanna\Desktop\PLOS REBUTTAL  AND REVISION\PLOSANALYS
IS2a.sav'
  /COMPRESSED.
EXAMINE VARIABLES=HOMAIR HOMAB
  /PLOT BOXPLOT
  /COMPARE GROUPS
  /STATISTICS DESCRIPTIVES EXTREME
  /CINTERVAL 95
  /MISSING LISTWISE
  /NOTOTAL.
```

Explore

Notes

Output Created		29-MAR-2019 15:48:29
Comments		
Input	Data	C:\Users\Prasanna\Desktop\PLOS REBUTTAL AND REVISION\PLOSANALYSIS2a.sav
	Active Dataset	DataSet1
	Filter	<none>
	Weight	<none>
	Split File	<none>
	N of Rows in Working Data File	12516
Missing Value Handling	Definition of Missing	User-defined missing values for dependent variables are treated as missing.
	Cases Used	Statistics are based on cases with no missing values for any dependent variable or factor used.
Syntax		EXAMINE VARIABLES=HOMAIR HOMAB<br>/PLOT BOXPLOT<br>/COMPARE GROUPS<br>/STATISTICS DESCRIPTIVES EXTREME<br>/CINTERVAL 95<br>/MISSING LISTWISE<br>/NOTOTAL.
Resources	Processor Time	00:00:01.09
	Elapsed Time	00:00:01.05

Case Processing Summary

	Cases					
	Valid		Missing		Total	
	N	Percent	N	Percent	N	Percent
HOMAIR	12516	100.0%	0	0.0%	12516	100.0%
HOMAB	12516	100.0%	0	0.0%	12516	100.0%

Descriptives

			Statistic	Std. Error
HOMAIR	Mean		3.5114	.04315
	95% Confidence Interval for Mean	Lower Bound	3.4268	
		Upper Bound	3.5959	
	5% Trimmed Mean		2.8395	
	Median		2.3151	
	Variance		23.308	
	Std. Deviation		4.82789	
	Minimum		.12	
	Maximum		98.89	
	Range		98.77	
	Interquartile Range		2.36	
	Skewness		7.478	.022
	Kurtosis		86.119	.044
HOMAB	Mean		146.2234	1.22830
	95% Confidence Interval for Mean	Lower Bound	143.8157	
		Upper Bound	148.6310	
	5% Trimmed Mean		128.6681	
	Median		110.8710	
	Variance		18883.176	
	Std. Deviation		137.41607	
	Minimum		1.02	
	Maximum		1998.93	
	Range		1997.91	
	Interquartile Range		102.73	
	Skewness		4.535	.022
	Kurtosis		35.056	.044

Extreme Values

			Case Number	Value
HOMAIR	Highest	1	12109	98.89
		2	11274	92.14
		3	12458	86.79
		4	383	80.55
		5	11307	78.07
	Lowest	1	1563	.12
		2	944	.12
		3	374	.13
		4	181	.13
		5	98	.14
HOMAB	Highest	1	12516	1998.93
		2	12515	1946.94
		3	12514	1935.00
		4	12513	1919.84
		5	12512	1896.26
	Lowest	1	1	1.02
		2	2	1.12
		3	3	1.74
		4	4	1.83
		5	5	2.43

HOMAIR

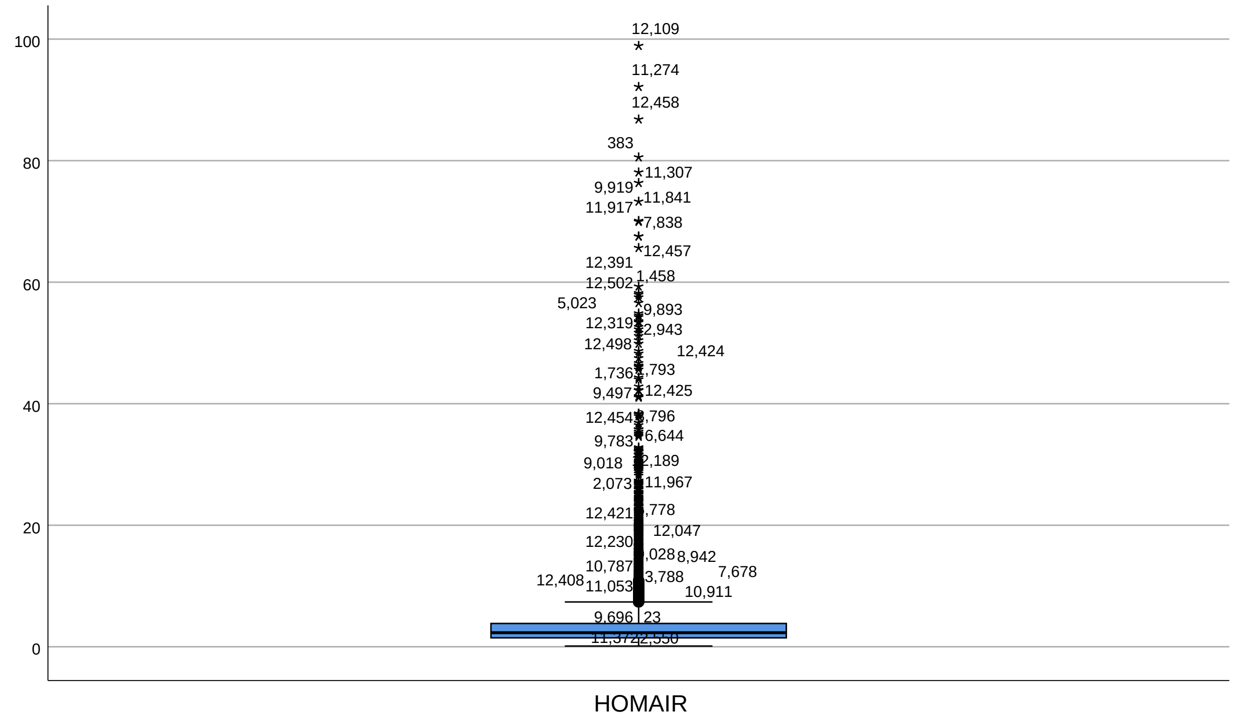

HOMAB

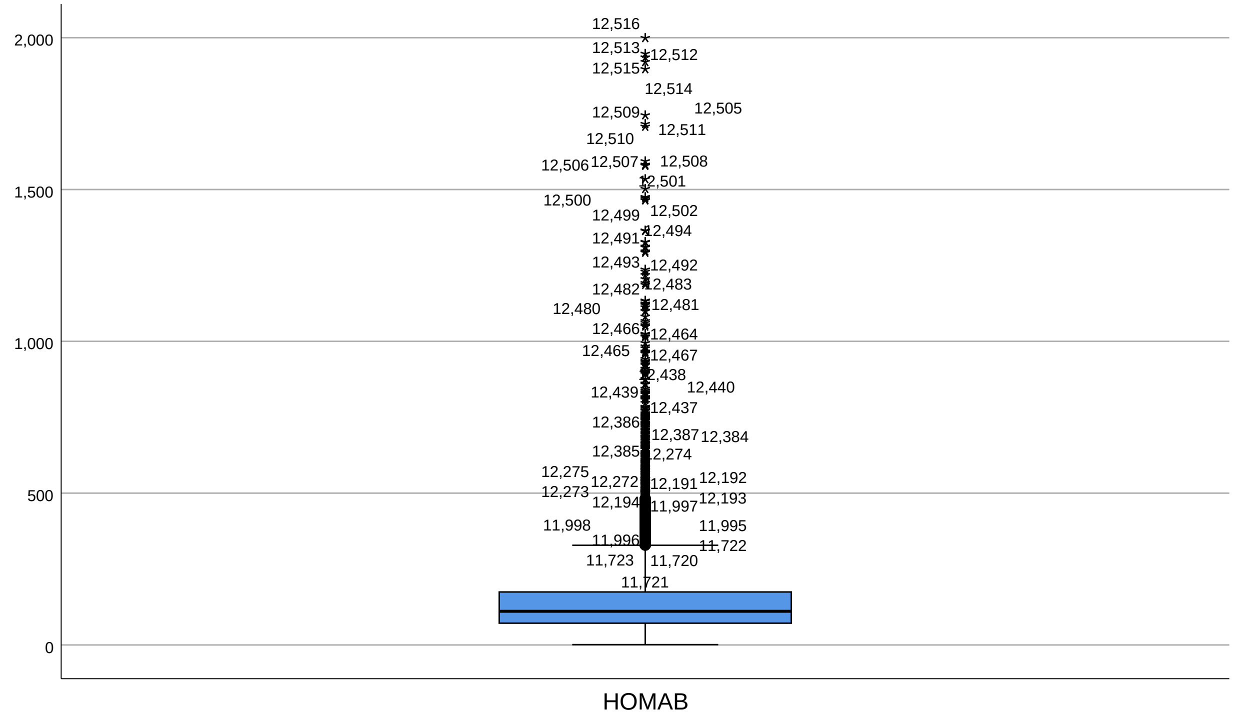

```
 COMPUTE LOGHOMAIR=LN(HOMAIR).
EXECUTE.
COMPUTE LOGHOMAB=LN(HOMAB).
EXECUTE.
DATASET ACTIVATE DataSet1.

SAVE OUTFILE='C:\Users\Prasanna\Desktop\PLOS REBUTTAL  AND REVISION\PLOSANALYS
IS2a.sav'
  /COMPRESSED.
EXAMINE VARIABLES=LOGHOMAIR LOGHOMAB
  /PLOT BOXPLOT HISTOGRAM NPPLOT
  /COMPARE GROUPS
  /STATISTICS DESCRIPTIVES EXTREME
  /CINTERVAL 95
  /MISSING LISTWISE
  /NOTOTAL.
```

Explore

Notes

Output Created		29-MAR-2019 15:51:36
Comments		
Input	Data	C: \Users\Prasanna\Desktop\ PLOS REBUTTAL AND REVISION\PLOSANALYS IS2a.sav
	Active Dataset	DataSet1
	Filter	<none>
	Weight	<none>
	Split File	<none>
	N of Rows in Working Data File	12516
Missing Value Handling	Definition of Missing	User-defined missing values for dependent variables are treated as missing.
	Cases Used	Statistics are based on cases with no missing values for any dependent variable or factor used.

Notes

Syntax		EXAMINE VARIABLES=LOGHOMAIR LOGHOMAB<br>/PLOT BOXPLOT HISTOGRAM NPPLOT<br>/COMPARE GROUPS<br>/STATISTICS DESCRIPTIVES EXTREME<br>/CINTERVAL 95<br>/MISSING LISTWISE<br>/NOTOTAL.
Resources	Processor Time	00:00:04.20
	Elapsed Time	00:00:03.89

Case Processing Summary

	Cases					
	Valid		Missing		Total	
	N	Percent	N	Percent	N	Percent
LOGHOMAIR	12516	100.0%	0	0.0%	12516	100.0%
LOGHOMAB	12516	100.0%	0	0.0%	12516	100.0%

Descriptives

			Statistic	Std. Error
LOGHOMAIR	Mean		.8894	.00707
	95% Confidence Interval for Mean	Lower Bound	.8755	
		Upper Bound	.9032	
	5% Trimmed Mean		.8731	
	Median		.8395	
	Variance		.626	
	Std. Deviation		.79150	
	Minimum		-2.13	
	Maximum		4.59	
	Range		6.72	
	Interquartile Range		.96	
	Skewness		.376	.022
	Kurtosis		1.255	.044
LOGHOMAB	Mean		4.7127	.00655
	95% Confidence Interval for Mean	Lower Bound	4.6998	
		Upper Bound	4.7255	
	5% Trimmed Mean		4.7170	
	Median		4.7084	
	Variance		.537	
	Std. Deviation		.73281	
	Minimum		.02	
	Maximum		7.60	
	Range		7.58	
	Interquartile Range		.89	
	Skewness		-.162	.022
	Kurtosis		1.532	.044

Extreme Values

			Case Number	Value
LOGHOMAIR	Highest	1	12109	4.59
		2	11274	4.52
		3	12458	4.46
		4	383	4.39
		5	11307	4.36
	Lowest	1	1563	-2.13
		2	944	-2.11
		3	374	-2.06
		4	181	-2.00
		5	98	-1.93
LOGHOMAB	Highest	1	12516	7.60
		2	12515	7.57
		3	12514	7.57
		4	12513	7.56
		5	12512	7.55
	Lowest	1	1	.02
		2	2	.11
		3	3	.55
		4	4	.60
		5	5	.89

Tests of Normality

	Kolmogorov-Smirnov[a]		
	Statistic	df	Sig.
LOGHOMAIR	.038	12516	.000
LOGHOMAB	.031	12516	.000

a. Lilliefors Significance Correction

LOGHOMAIR

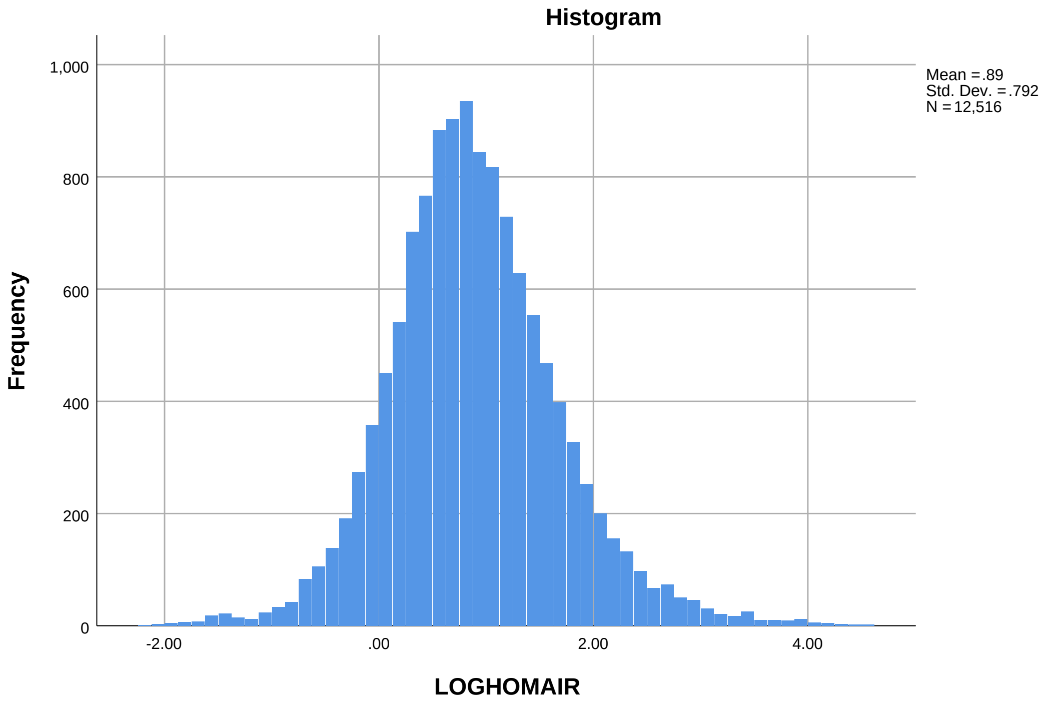
Histogram
Mean =.89
Std. Dev. =.792
N =12,516
Frequency
1,000
800
600
400
200
0
-2.00
.00
2.00
4.00
LOGHOMAIR

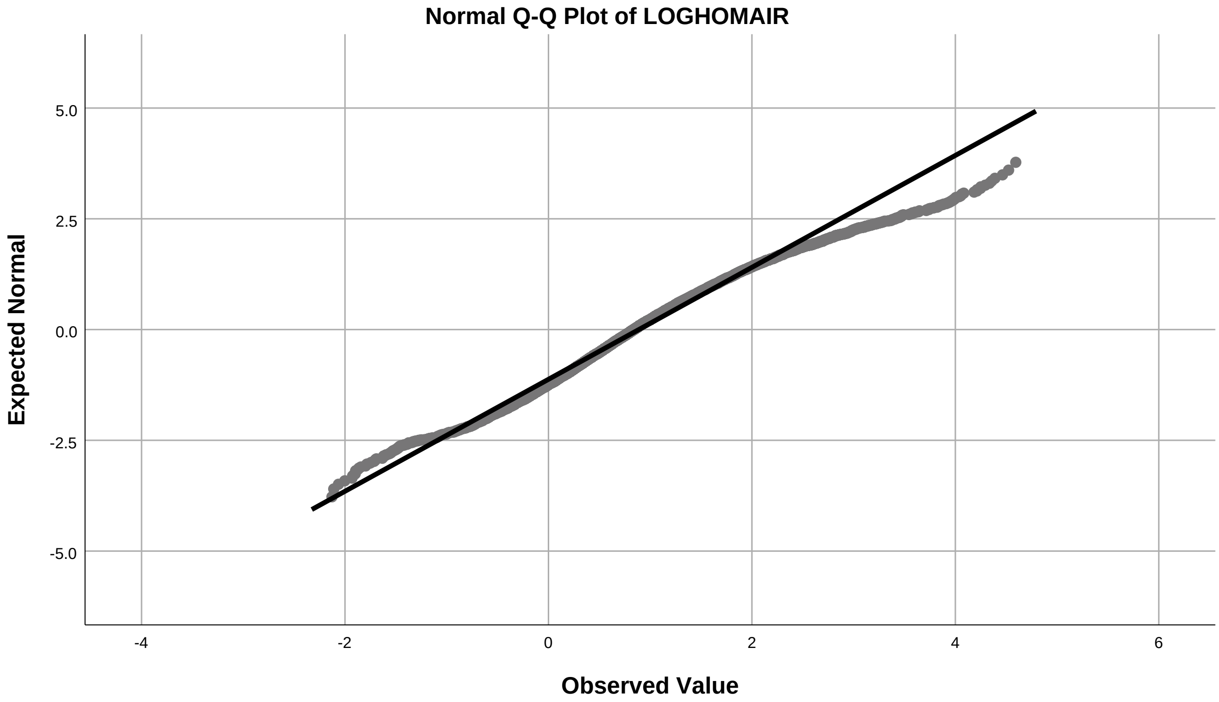
Normal Q-Q Plot of LOGHOMAIR
Expected Normal
5.0
2.5
0.0
-2.5
-5.0
-4
-2
0
2
4
6
Observed Value

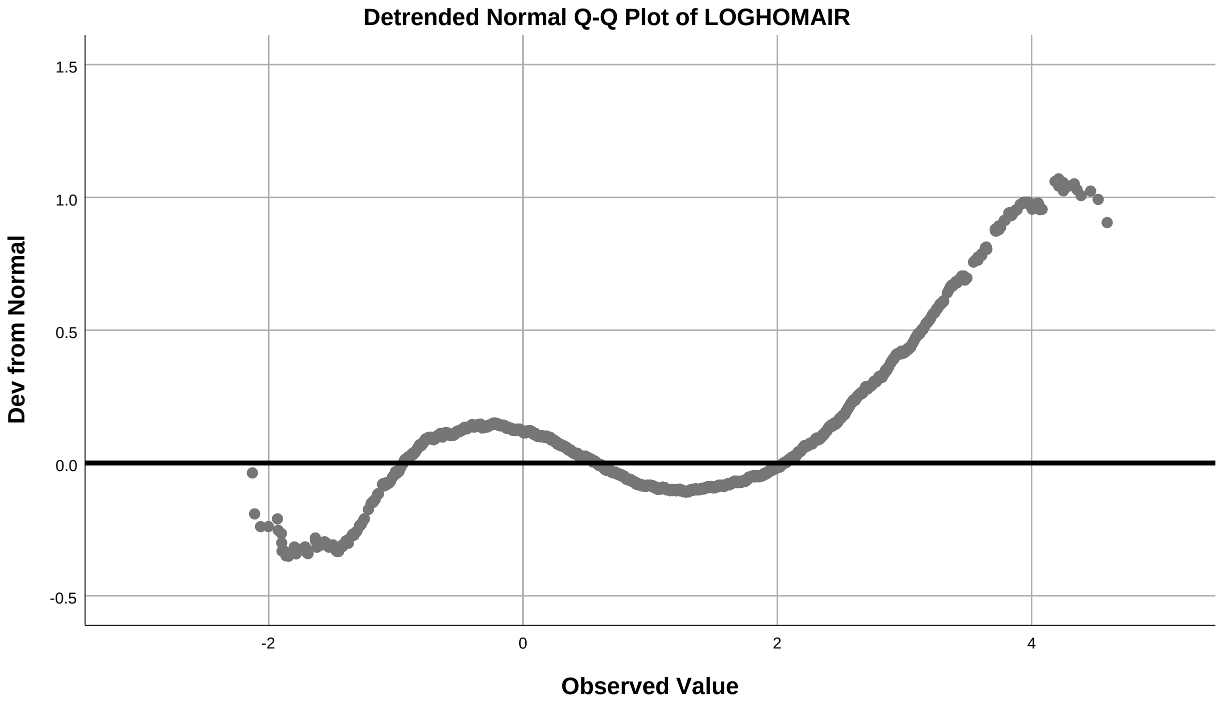
Detrended Normal Q-Q Plot of LOGHOMAIR
Dev from Normal
Observed Value
1.5
1.0
0.5
0.0
-0.5
-2
0
2
4

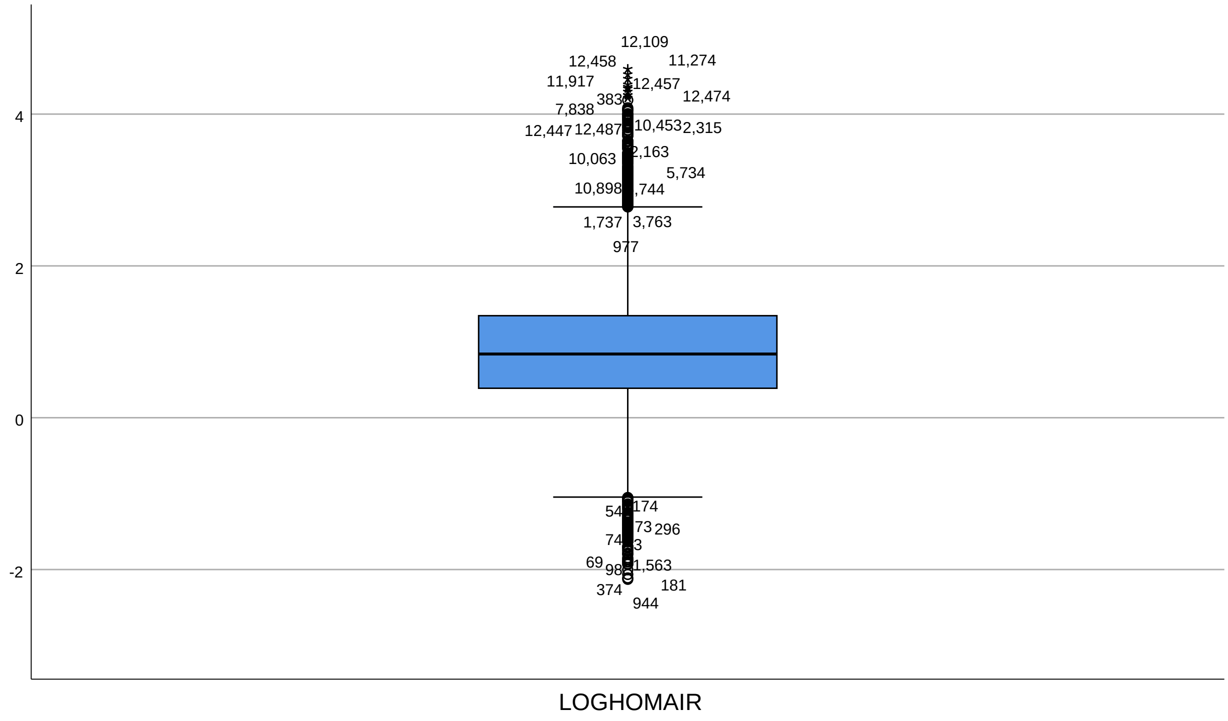
LOGHOMAIR
4
2
0
-2

LOGHOMAB

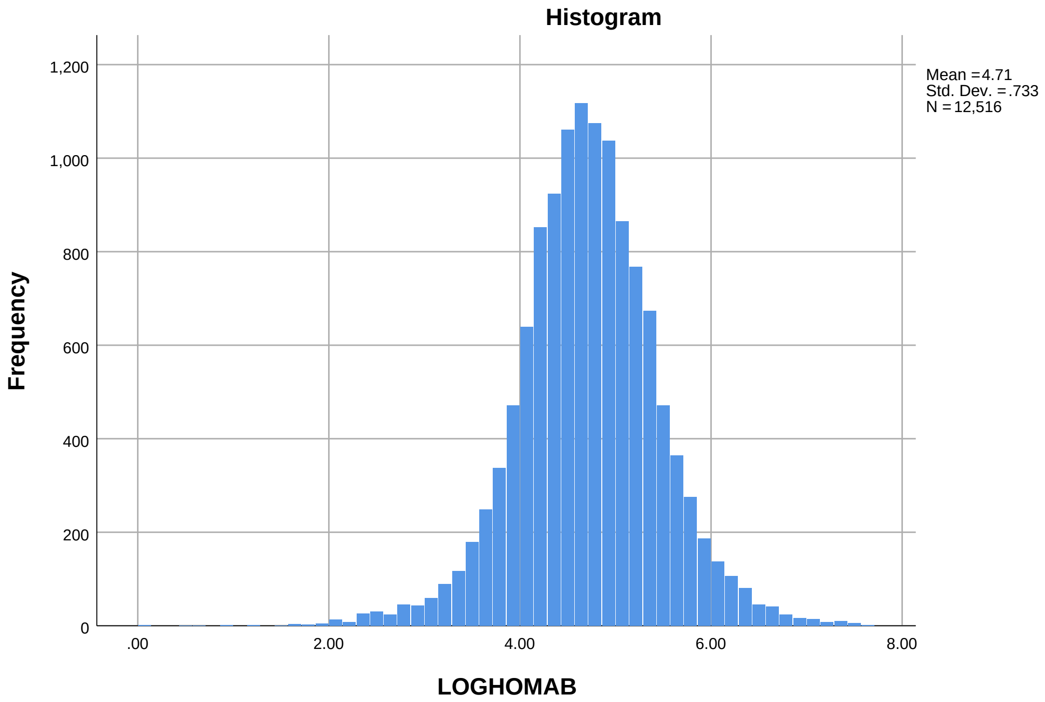
Histogram
Mean =4.71
Std. Dev. =.733
N =12,516
Frequency
1,200
1,000
800
600
400
200
0
.00
2.00
4.00
6.00
8.00
LOGHOMAB

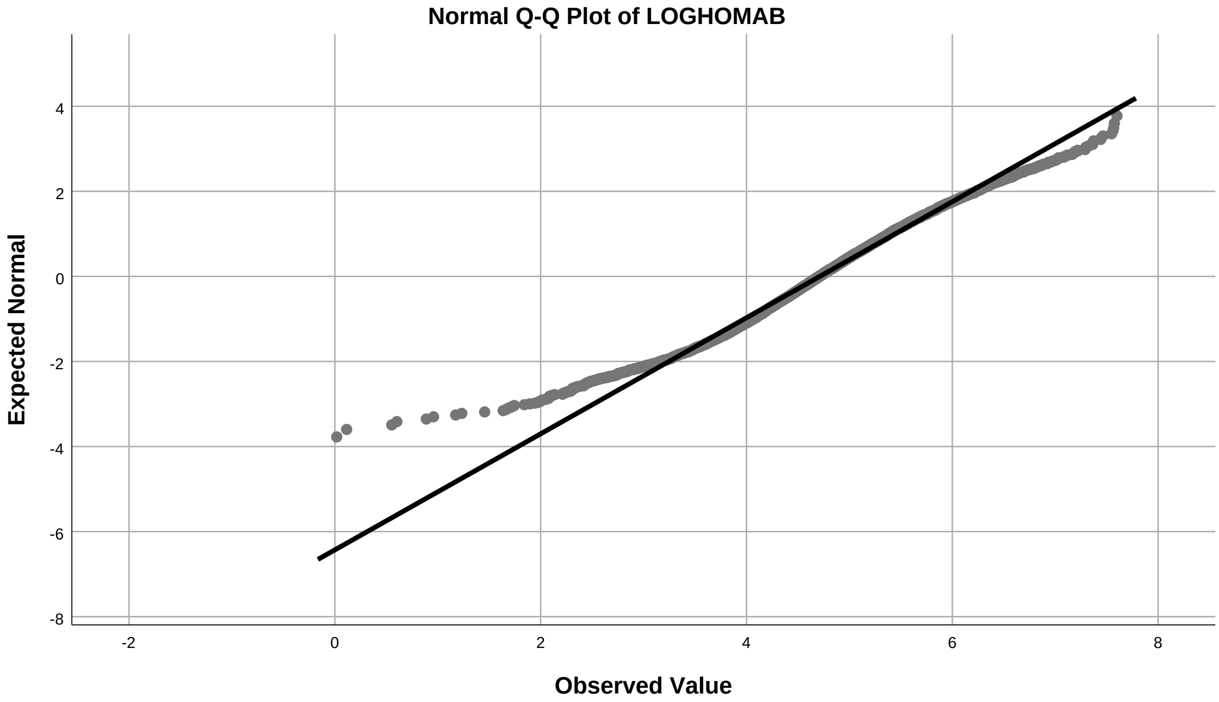
Normal Q-Q Plot of LOGHOMAB
Expected Normal
4
2
0
-2
-4
-6
-8
-2
0
2
4
6
8
Observed Value

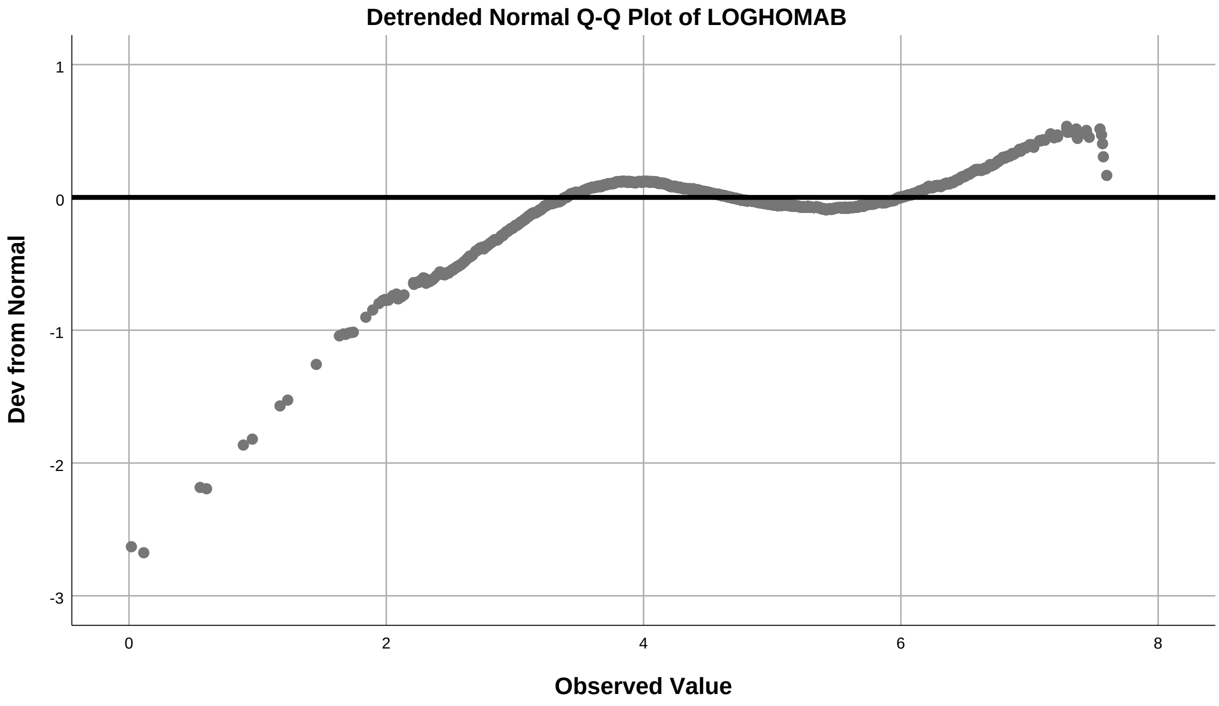

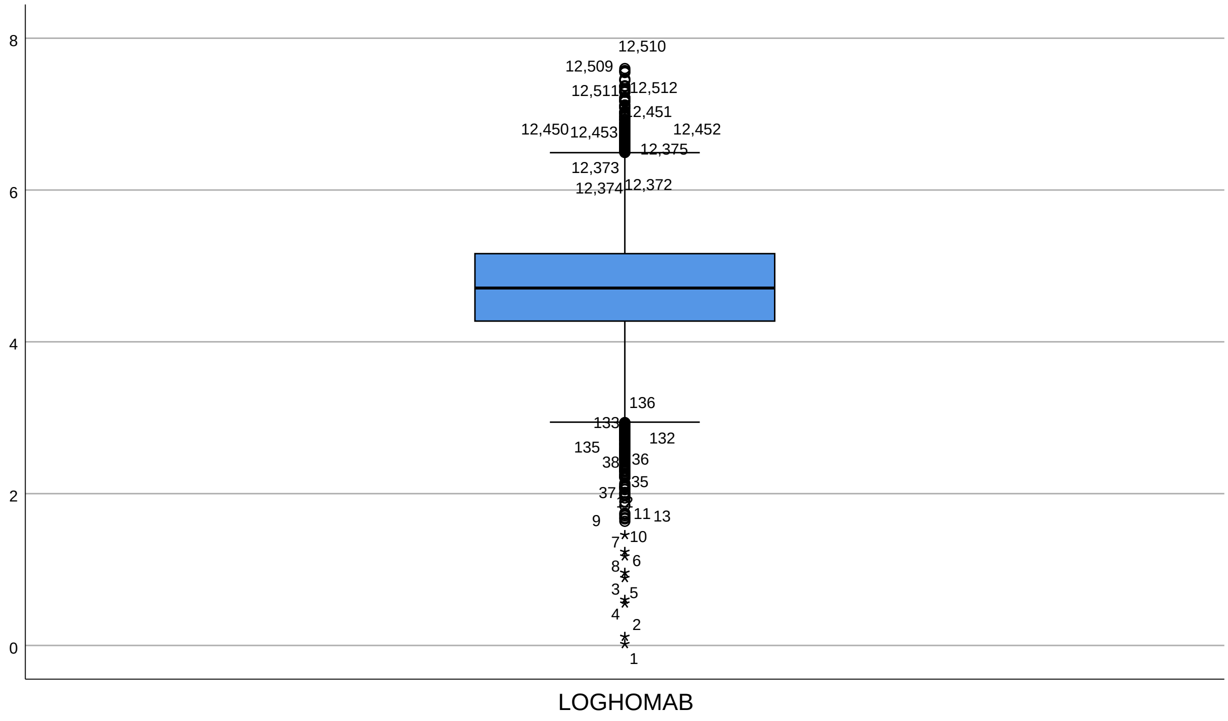


```
COMPUTE LOG10HOMAIR=LG10(HOMAIR).
EXECUTE.
```

```
COMPUTE LOG10HOMAB=LG10(HOMAB).
EXECUTE.
DATASET ACTIVATE DataSet1.

SAVE OUTFILE='C:\Users\Prasanna\Desktop\PLOS REBUTTAL  AND REVISION\PLOSANALYS
IS2a.sav'
  /COMPRESSED.
EXAMINE VARIABLES=LOG10HOMAIR LOG10HOMAB
  /PLOT HISTOGRAM NPPLOT
  /STATISTICS DESCRIPTIVES EXTREME
  /CINTERVAL 95
  /MISSING LISTWISE
  /NOTOTAL.
```

Explore

Notes

Output Created		29-MAR-2019 15:57:29
Comments		
Input	Data	C:\Users\Prasanna\Desktop\PLOS REBUTTAL AND REVISION\PLOSANALYSIS2a.sav
	Active Dataset	DataSet1
	Filter	<none>
	Weight	<none>
	Split File	<none>
	N of Rows in Working Data File	12516
Missing Value Handling	Definition of Missing	User-defined missing values for dependent variables are treated as missing.
	Cases Used	Statistics are based on cases with no missing values for any dependent variable or factor used.

Notes

Syntax		EXAMINE VARIABLES=LOG10HOMAIR LOG10HOMAB<br>/PLOT HISTOGRAM NPPLOT<br>/STATISTICS DESCRIPTIVES EXTREME<br>/CINTERVAL 95<br>/MISSING LISTWISE<br>/NOTOTAL.
Resources	Processor Time	00:00:03.27
	Elapsed Time	00:00:02.94

Case Processing Summary

	Cases					
	Valid		Missing		Total	
	N	Percent	N	Percent	N	Percent
LOG10HOMAIR	12516	100.0%	0	0.0%	12516	100.0%
LOG10HOMAB	12516	100.0%	0	0.0%	12516	100.0%

Descriptives

			Statistic	Std. Error
LOG10HOMAIR	Mean		.3862	.00307
	95% Confidence Interval for Mean	Lower Bound	.3802	
		Upper Bound	.3923	
	5% Trimmed Mean		.3792	
	Median		.3646	
	Variance		.118	
	Std. Deviation		.34375	
	Minimum		-.92	
	Maximum		2.00	
	Range		2.92	
	Interquartile Range		.42	
	Skewness		.376	.022
	Kurtosis		1.255	.044
LOG10HOMAB	Mean		2.0467	.00284
	95% Confidence Interval for Mean	Lower Bound	2.0411	
		Upper Bound	2.0523	
	5% Trimmed Mean		2.0486	
	Median		2.0448	
	Variance		.101	
	Std. Deviation		.31826	
	Minimum		.01	
	Maximum		3.30	
	Range		3.29	
	Interquartile Range		.39	
	Skewness		-.162	.022
	Kurtosis		1.532	.044

Extreme Values

			Case Number	Value
LOG10HOMAIR	Highest	1	12109	2.00
		2	11274	1.96
		3	12458	1.94
		4	383	1.91
		5	11307	1.89
	Lowest	1	1563	-.92
		2	944	-.92
		3	374	-.90
		4	181	-.87
		5	98	-.84
LOG10HOMAB	Highest	1	12516	3.30
		2	12515	3.29
		3	12514	3.29
		4	12513	3.28
		5	12512	3.28
	Lowest	1	1	.01
		2	2	.05
		3	3	.24
		4	4	.26
		5	5	.39

Tests of Normality

	Kolmogorov-Smirnov[a]		
	Statistic	df	Sig.
LOG10HOMAIR	.038	12516	.000
LOG10HOMAB	.031	12516	.000

a. Lilliefors Significance Correction

LOG10HOMAIR

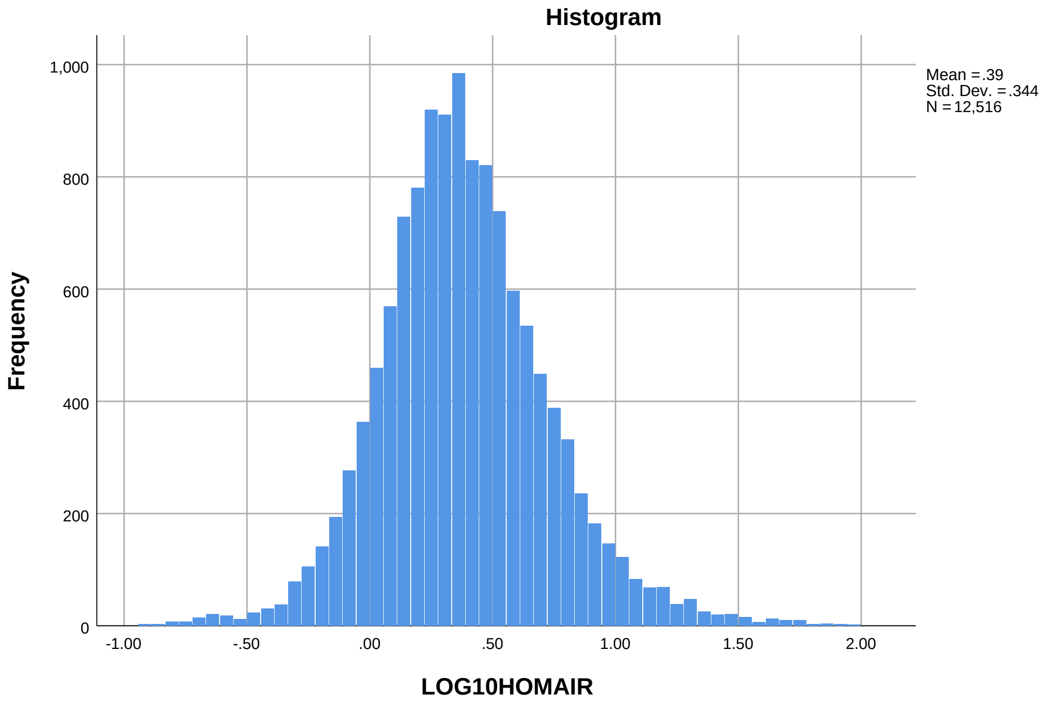
Histogram
Frequency
LOG10HOMAIR
Mean =.39
Std. Dev. =.344
N =12,516
1,000
800
600
400
200
0
-1.00
-.50
.00
.50
1.00
1.50
2.00

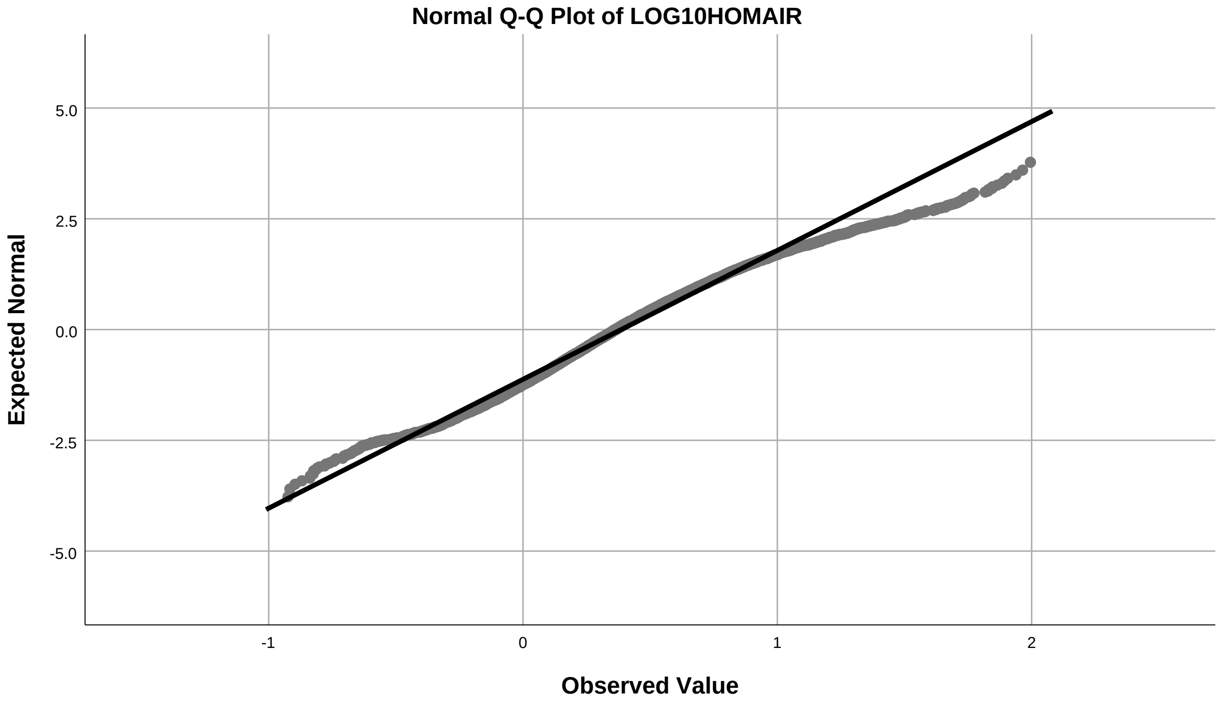
Normal Q-Q Plot of LOG10HOMAIR
Expected Normal
Observed Value
5.0
2.5
0.0
-2.5
-5.0
-1
0
1
2

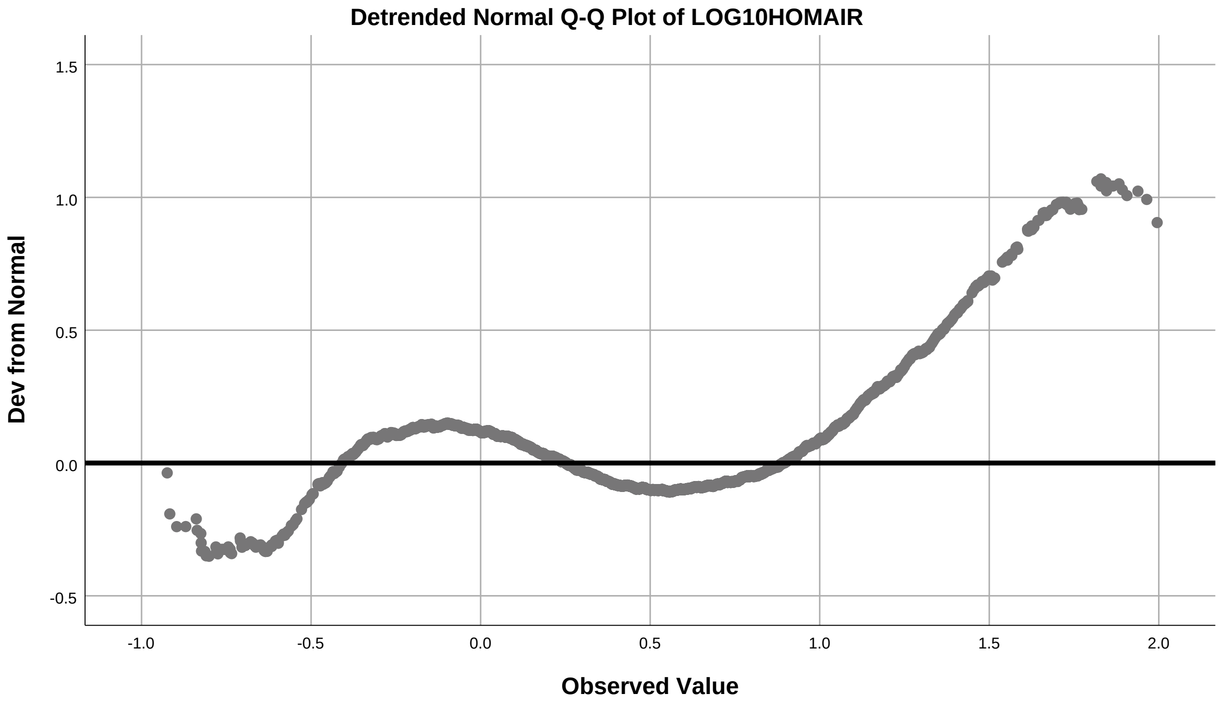
Detrended Normal Q-Q Plot of LOG10HOMAIR
Dev from Normal
Observed Value
1.5
1.0
0.5
0.0
-0.5
-1.0
-0.5
0.0
0.5
1.0
1.5
2.0

LOG10HOMAB

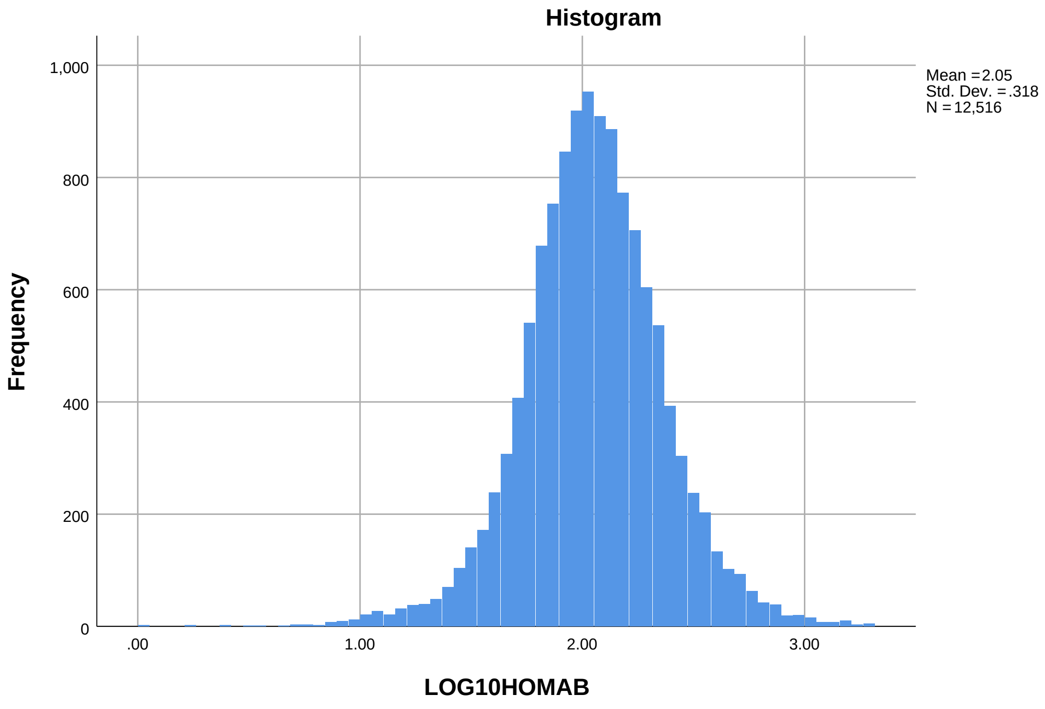
Histogram
Frequency
LOG10HOMAB
Mean =2.05
Std. Dev. =.318
N =12,516
1,000
800
600
400
200
0
.00
1.00
2.00
3.00

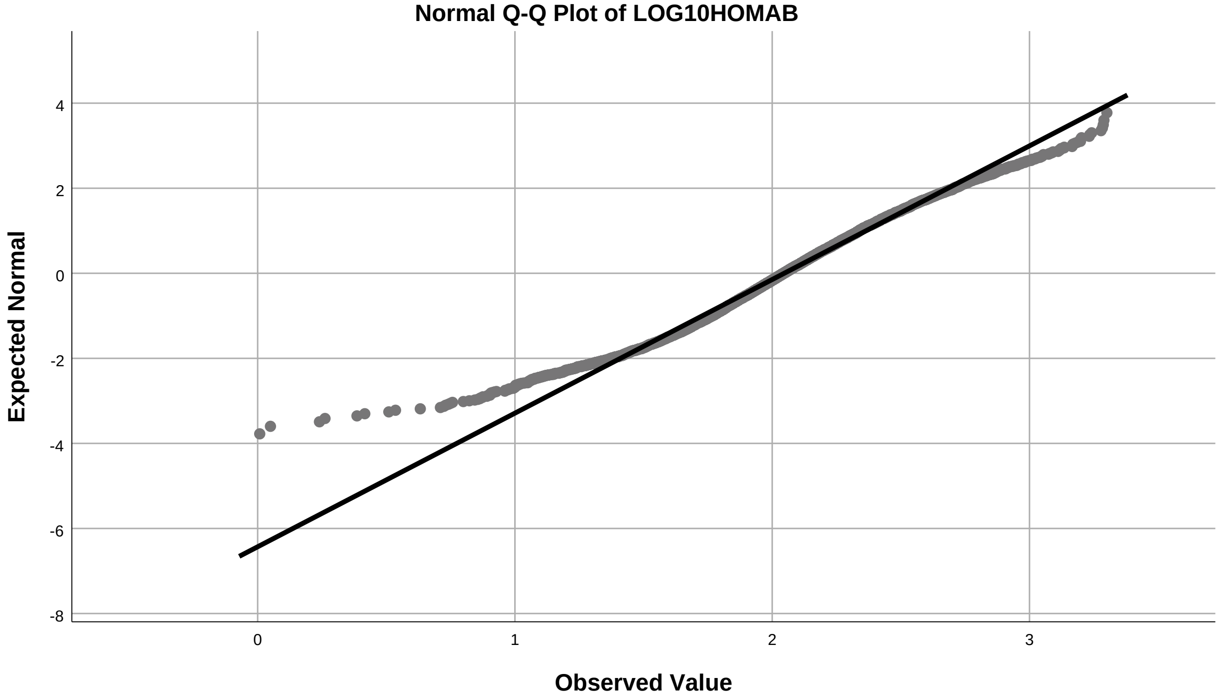
Normal Q-Q Plot of LOG10HOMAB
Expected Normal
4
2
0
-2
-4
-6
-8
0
1
2
3
Observed Value

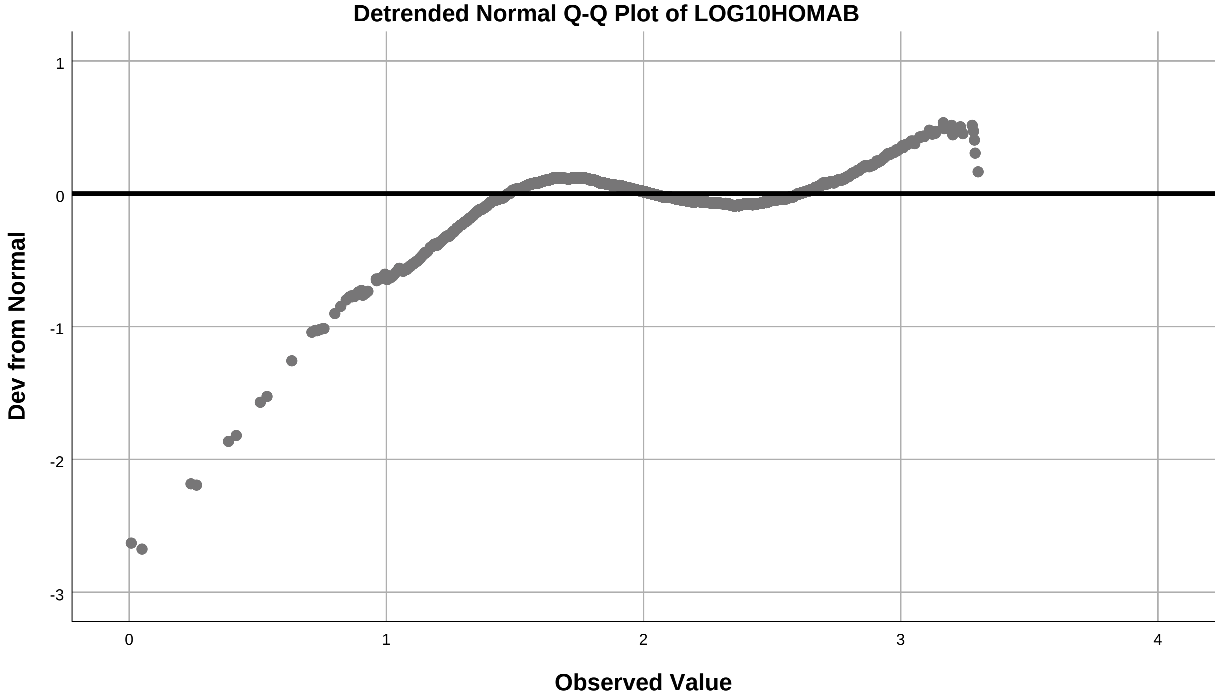
Detrended Normal Q-Q Plot of LOG10HOMAB
Dev from Normal
1
0
-1
-2
-3
0
1
2
3
4
Observed Value
